# Direct Readout of Homo- vs Heterochiral Ligand Shell of Quantum Dots

**DOI:** 10.1021/acsami.4c07648

**Published:** 2024-07-08

**Authors:** Elżbieta Chwojnowska, Aneta A. Kowalska, Agnieszka Kamińska, Janusz Lewiński

**Affiliations:** † Institute of Physical Chemistry Polish Academy of Sciences, Kasprzaka 44/52 , Warsaw 01-224, Poland; ‡ Faculty of Chemistry, Warsaw University of Technology, Noakowskiego 3 , Warsaw 00-664, Poland

**Keywords:** quantum dots, chiral environment, mixed ligand shell, zinc oxide, Raman spectroscopy, principal component analysis

## Abstract

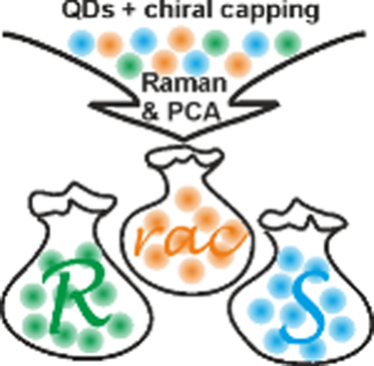

The chiroptical activity of various semiconductor inorganic nanocrystalline materials has typically been tested using circular dichroism or circularly polarized luminescence. Herein, we report on a high-throughput screening method for identifying and differentiating chiroptically active quantum-sized ZnO crystals using Raman spectroscopy combined with principal component analysis. ZnO quantum dots (QDs) coated by structurally diverse homo- and heterochiral aminoalcoholate ligands (*cis*- and *trans*-1-amino-2-indanolate, 2-amino-1-phenylethanolate, and diphenyl-2-pyrrolidinemethanolate) were prepared using the one-pot self-supporting organometallic procedure and then extensively studied toward the identification of specific Raman fingerprints and spectral variations. The direct comparison between the spectra demonstrates that it is very difficult to make definite recognition and identification between QDs coated with enantiomers based only on the differences in the respective Raman bands’ position shifts and their intensities. However, the applied approach involving the principal component analysis performed on the Raman spectra allows the simultaneous differentiation and identification of the studied QDs. The first and second principal components explain 98, 97, 97, and 87% of the variability among the studied families of QDs and demonstrate the possibility of using the presented method as a qualitative assay. Thus, the reported multivariate approach paves the way for simultaneous differentiation and identification of chirotopically active semiconductor nanocrystals.

## 1 Introduction

Solution-processable semiconductor nanocrystals (NCs), known also as colloidal quantum dots (QDs),^1^ with a wide range of unique size-, shape-, and composition-dependent physicochemical properties, are fundamental to modern science and technology.^2^ Over three decades ago, Louis E. Brus—one of the pioneers of nanochemistry and colloidal QDs—stated that “the enormity of this project is obvious, yet an encouraging start has been made”.^3^ Indeed, QDs are an important research area in nanoscience and nanotechnology and while the research in the field is constantly moving forward, there are a number of challenges.^4^ For example, detailed characterization of the ligand shell composition is required to optimize NC properties and surface interactions for a vast array of applications.^5−9^ Transferring chirality from enantiomeric molecules to colloidal QDs represents another level of tailorability and has attracted immense attention across chemistry, materials, and biomedical science.^10,11^ Chiroptical properties of NCs arouse interest at the level of basic research,^12,13^ as well as in the context of various applications, including photonics, catalysis, sensing, and biomedicine.^14−18^ For instance, nanomaterials fabricated using chiral ligands have aroused substantial interest due to the special chirality-dependent biological effects, and a recent seminal study demonstrated that nanoscale chirality can be exploited to modulate immunological responses^19^ and various types of chiral nanoparticles have been explored in cancer therapy.^20^ Hence, the development of chiroptically active colloidal QDs and understanding of their optical activity have been a vital issue of nanoresearch over the past decade^21,22^ following the first observation of chiroptical activity in CdS QDs prepared by microwave-assisted synthesis in the presence of l- or d-penicillamine by Moloney et al. in 2007.^23^ This area of research has been dominated by heavy metal-based QDs.^24−26^ Commonly used protocol for the preparation of metal-based QDs involves postsynthetic ligand replacement by chiral molecules after the achiral pristine particle synthesis has been completed. Nevertheless, while adjusting the chirality of NCs, this approach involving ligand exchange reactions has many disadvantages and, for example, may induce uncontrollable changes to the NC surface upon ligand exchange and ultimately, in many cases, lead to the formation of mixed ligand shells.^27,28^

The chiroptical activity of QDs is typically tested using electronic circular dichroism,^24,25^ or circularly polarized luminescence.^29,30^ In turn, Raman spectroscopy has been used as a powerful method to investigate different properties of nanocrystalline materials (including ZnO) such as composition,^31^ crystallite size distribution,^32,33^ disorder and thickness of the ligand shell,^34^ and changes associated with doping^35^ or to calculate the surface and interface parameters.^36,37^ However, to the best of our knowledge, this technique has not been used to differentiate between QDs coated exclusively by single enantiomers and QDs possessing the organic shell composed of a racemic mixture of these enantiomers, even though number of studies of various chiral biologically important entities (e.g., sugars, DNA, proteins, molecules) have demonstrated that Raman spectroscopy (including surface-enhanced Raman spectroscopy,^38,39^ which provides a way to detect smaller quantities) is a very promising and powerful technique, offering rapid sample screening to distinguish the respective enantiomeric forms.^40−42^ A critical part of the differentiation strategy based on Raman spectroscopy is the identification of both significant bands and a proper algorithm that can analyze the measured data set.^43−45^ In this regard, principal component analysis (PCA), as a statistical method, is useful for finding a pattern in Raman data of high dimensions. The efficiency of PCA relies on the ability to transform high-complexity Raman data into a new coordinate principal component (PC) system using an orthogonal linear transformation with the axes oriented to show the maximal variation in the data set.^46,47^ A dozen scientific papers show the value of PCA, especially in hyperspectral mapping, characterization, detection, identification, and distribution approaches.^48−50^ As we have mentioned before, the introduction of chiral ligands has been regarded as an effective strategy to obtain nanoclusters with optical purity. Access to such a variety of NCs could be particularly useful in potential investigations on enantiomer-dependent immunological responses to chiral nanoparticles or studies directed to a better understanding of the origin of chiroptical activity in nanostructures.

Interfacing QDs with chiral-ligand-based surface functionalization, including racemic ligand systems, add another level of complexity, making it difficult to probe the combined system adequately using existing techniques. Herein, we identify specific Raman fingerprints and spectral variations for a model series of ZnO QDs coated with structurally diverse homochiral and heterochiral organic ligands and apply Raman spectroscopy combined with PCA to elaborate a method that allows differentiation between QDs with chiral and racemic shell as well as between QDs of different handedness.

Reports on chiral ZnO nanostructures include various structures like nanosprings, nanospirals, nanohelixes,^51,52^ or chiral films,^53,54^ whereas studies on chiroptically active colloidal ZnO QDs are still scarce. For example, l- and d-cysteine-^55,56^ and l- and d-arginine-coated ZnO QDs^57^ were prepared by a modified sol–gel method using chiral molecules as a surface stabilizer during synthesis. However, it should be noted that the conventional sol–gel procedure is an attractive synthetic approach yet uncontrollable process leading to QDs with an ill-passivated and unstable surface and a complicated heterogeneous coating shell composed.^58−60^ Significant progress to the field has been made thanks to the one-pot self-supporting organometallic (OSSOM) procedure, a general synthetic method based on the controlled exposure of [RZn(X)]n-type (X = monoanionic organic ligand) precursors to air at ambient temperature.^58,59^ The OSSOM approach allowed the preparation of a series of QDs with both a homochiral organic shell composed of strongly anchored enantiomerically pure aminoalcoholate capping ligands, and subnanometer control of size with diameters below 10 nm that are well suited for investigating size-dependent optical properties.^61^ The chiroptical responses originated from the multipoint interactions of aminoalcoholate ligands with the surface through the amine nitrogen and the alcoholate oxygen that can transmit an enantiomeric structural imprint on the ZnO surface. Herein, we selected a pool of enantiomerically pure aminoalcohols and racemic mixtures of those aminoalcohols and prepared a vast array of homo- and heterochiral ligand-coated QDs using the OSSOM procedure. Then, the resulting landscape of QDs was tested as a model system for discrimination of the homo- and heterochiral ligand-coated QDs using Raman spectroscopy combined with PCA as a direct probe.

## 2 Results and Discussion

### 2.1 Synthesis of ZnO QDs

**Figure 1:**
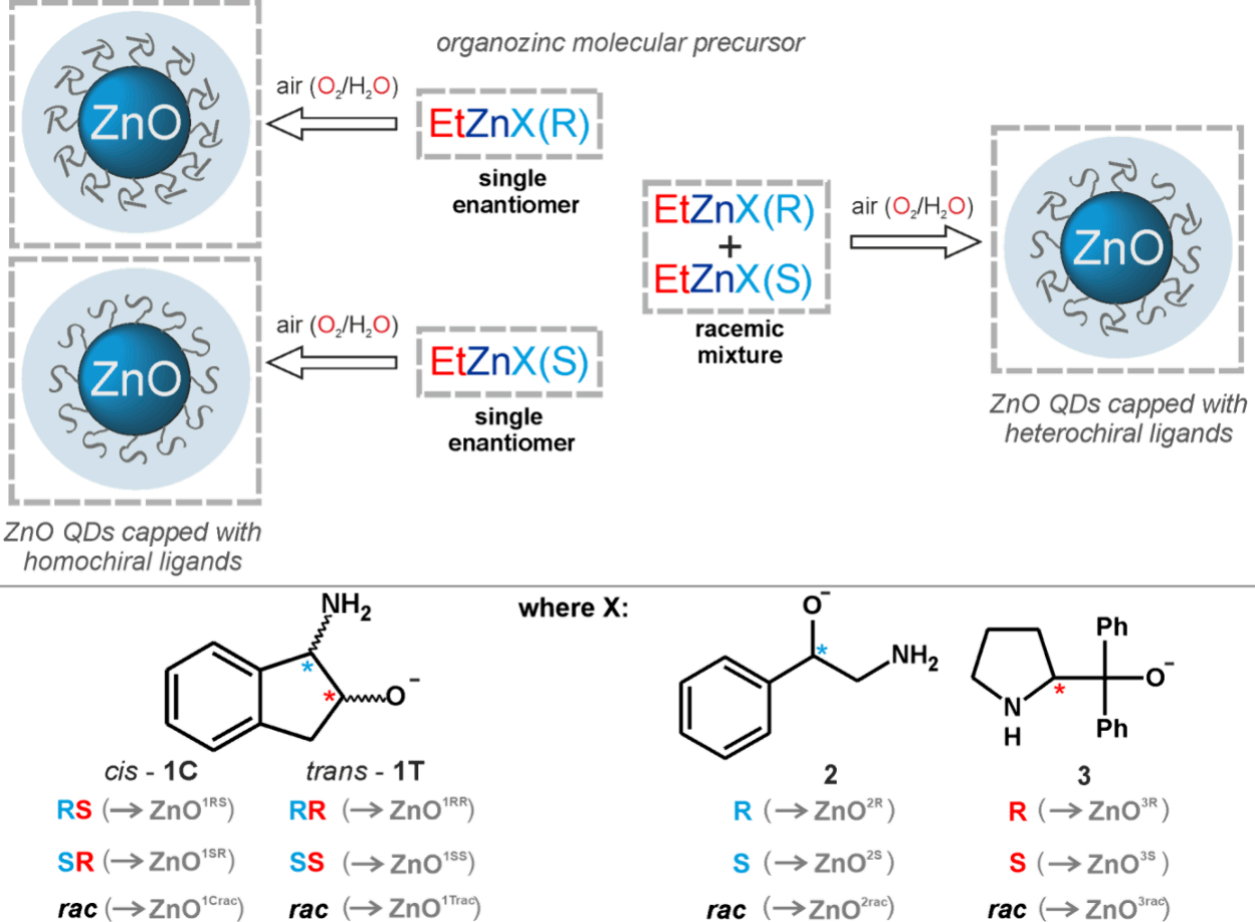
Schematic representation of the self-supporting organometallic (OSSOM) procedure for the preparation of ZnO QDs capped with homochiral or heterochiral ligands.

For the purpose of this study, we extended the pool of proligands from previously used enantiomerically pure (1*S*,2*R*)- and (1*R*,2*S*)-*cis*-1-amino-2-indanol, *S*- and *R*-amino-1-phenylethanol, *S*- and *R*-α,α-diphenyl-2-pyrrolidinemethanol to (1*S*,2*S*)- and (1*R*,2*R*)-trans-1-amino-2-indanol and additionally harnessed racemic mixtures of those aminoalcohols (Figure 1).

Rational selection of alkyl zinc molecular precursors incorporating chiral aminoalcoholate ligands in the OSSOM procedure ensures both QDs with alkoxide ligands firmly anchored to the surface and chirality transfer from the ligands to the inorganic core–ligand interface during the QD synthesis.^61^ Thus, in the first step, [EtZn(X)]-type organozinc precursors were obtained in the reaction of Et_2_Zn with the respective amino alcohol as an X–H proligand. Then, *in situ* generated [EtZn(X)]-type precursors were exposed to air to afford a landscape of ZnO QDs coated with chiral or racemic organic shells, respectively (for details, see Section 4.2). In this vein, we prepared 12 series-connected colloidal QDs that can be divided into four families defined by the character of aminoalcoholate ligands: (i) *cis*-aminoindanolate-coated QDs (hereinafter denoted as **ZnO**^**1C**^)—this family includes QDs capped by (1*R*, 2*S*)-, (1*S*, 2*R*)-, and *rac*-*cis*-1-amino-2-indanolate (**ZnO**^**1RS**^, **ZnO^1SR^**, and **ZnO**^**1C*****rac***^, respectively); (ii) *trans*-aminoindanolate-coated **ZnO**^**1T**^: (1*R*, 2*R*)-, (1*S*, 2*S*)-, and *rac*-*trans*-1-amino-2-indanolate-capped **ZnO**^**1RR**^, **ZnO^1SS^**, and **ZnO**^**1T*****rac***^, respectively; (iii) aminophenylethanolate-coated **ZnO**^**2**^: *R*-, *S*-, and *rac*-amino-1-phenylethanolate-capped **ZnO**^**2R**^, **ZnO**^**2S**^, and **ZnO**^**2*****rac***^, respectively; and (iv) diphenylpyrrolidinemethanolate-coated **ZnO**^**3**^: *R*-, *S*-, and *rac*-diphenyl-2-pyrrolidinemethanolate-capped **ZnO**^**3R**^, **ZnO**^**3S**^, and **ZnO**^**3*****rac***^, respectively. The resulting QDs differ not only by the coating organic ligand but also by the inorganic core size determined by the character of aminoalcoholate ligands. A close relationship between the size of the core and the X-type ligand used is a typical feature of the OSSOM procedure.^58,61,62^ All nanomaterials exhibit relatively low polydispersity, and the average sizes of the inorganic cores of the resulting QDs are about 1.7 nm for **ZnO**^**1C**^ and **ZnO**^**1T**^ and about 3 and 7 nm for **ZnO**^**2**^ and **ZnO**^**3**^, respectively (Figures S1–S18). Interestingly, the inorganic core sizes of **ZnO**^**1T**^ capped by *trans*-aminoindanolates are similar to that of **ZnO**^**1C**^ capped by *cis*-aminoindanolates (Figures S1–S10).

### 2.2 Characterization of Homo- vs Heterochiral Ligand Shell of QDs Using Raman Spectroscopy

In the next step, the families of **ZnO**^**1C**^, **ZnO**^**1T**^, **ZnO**^**2**^, and **ZnO**^**3**^ QDs were characterized using Raman spectroscopy to identify specific Raman fingerprints and spectral variations for each family. ZnO QDs crystallize in the wurtzite structure with the hexagonal C_*6v*_^4^(*P*6_3_mc) space group, and according to the group theory, the existence of the following optic modes is expected: Γ = A_1_ + 2B_1_ + E_1_ + 2E_2_. Both B_1_ (low) and B_1_ (high) modes are normally silent, while A and E modes are polar and split into transverse optical (TO) and longitudinal optical (LO) phonons.^63−65^ In relation to the selection rules, A_1_, E_1_, and E_2_ modes are Raman active while B_1_ is forbidden. All these Raman active phonon modes can be recognized as the characteristic bands of ZnO hexagonal wurtzite phase in the low wavenumber region (350–500 cm^–1^).^66^

**Figure 2:**
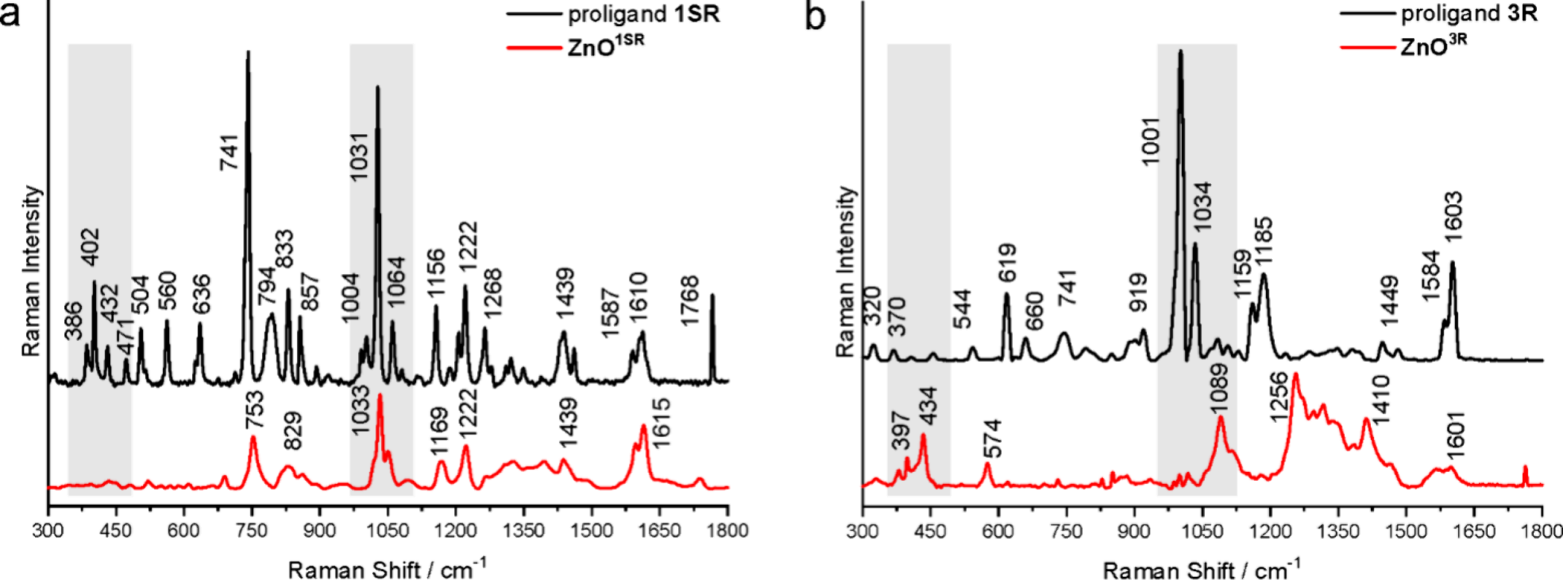
Raman spectra of **ZnO**^**1SR**^ and (1*S*,2*R*)-*cis-*1-amino-2-indanol (a) and **ZnO**^**3R**^ and *R*-diphenyl-2-pyrrolidinemethanol (b).

The spectra of both aminoalcoholate ligand-coated QDs and respective amino alcohol proligands (for comparison) were recorded in the 300–1800 cm^–1^ region (Figure 2 and Figure S21). The binding of aminoalcoholate ligands to the ZnO surface results in the substantial spectral differences due to the core–ligand interactions, and the most pronounced differences in the spectra of QDs and proligands are observed in the two regions, i.e., 350–500 and 950–1150 cm^–1^. The low wavenumber region is characteristic for the ZnO modes^33,66,67^ and thus is especially important for the analysis. The second region is dominated by the coating aminoalcoholate ligand vibrations^68,69^ and contains the most intensive bands observed for all studied ligands (Figure 2 and Figure S21), but in some instances, the ZnO combination modes and overtones are present.^33^ Generally, structurally diverse ligands bonded to the surface differently influence the force constants and vibrational amplitudes of the nearest-neighbor bonds, which directly implicates the changes in intensities of the bands related ligand bond vibrations. Thus, different spectral effects are expected for QDs with specific ligand shells. For example, for (1*S*,2*R*)-*cis-*1-amino-2-indanol, a large diversity of Raman bands in the region 350–500 cm^–1^ is observed (Figure 2a) and the analogous spectral features for other aminoindanols are noticed (Figure S21a,b). However, a different situation is noticed for the spectra of the respective QDs. In this spectral region, for **ZnO**^**1C**^ and **ZnO**^**1T**^, either only a few low-intensity bands are observed (Figure S21a,b) or no bands, as it is in the case of the **ZnO**^**1SR**^ (Figure 2a). This indicates that due to the core–ligand interactions, the overlapping of the less intense ZnO modes for **ZnO**^**1C**^ and **ZnO**^**1T**^, a so-called screening effect, appeared. In turn, only a few low-intensity bands are observed in the spectra of diphenyl-2-pyrrolidinemethanols and aminophenylethanols. Thus, in this case, a specific spectral window appears for detecting vibrational modes of the ZnO inorganic core in the **ZnO**^**2**^ and **ZnO**^**3**^ families (Figure 2b and Figure S21b,c).

**Figure 3:**
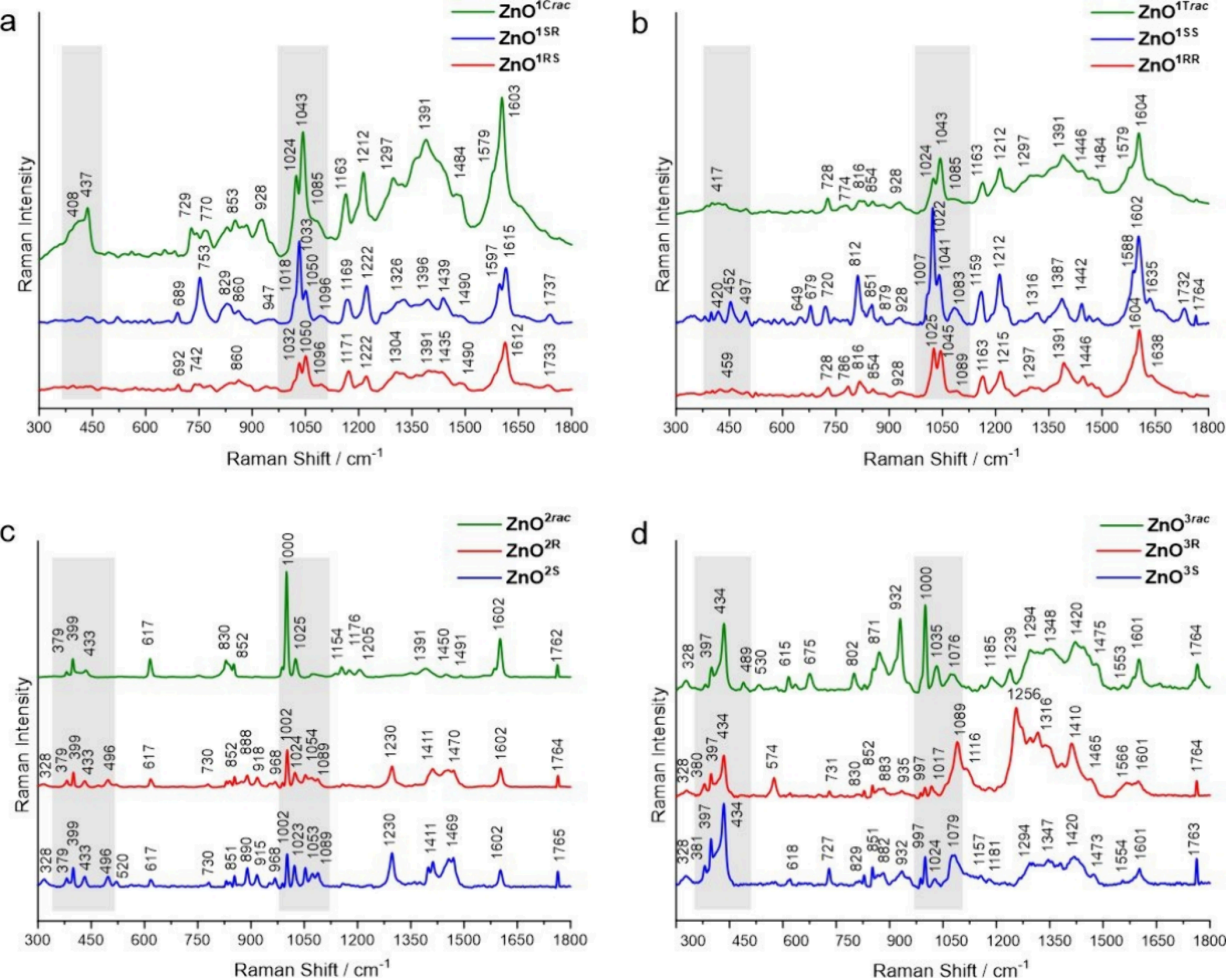
Raman spectra of **ZnO**^**1SR**^, **ZnO**^**1RS**^, and **ZnO**^**1C*****rac***^ (a); **ZnO**^**1SS**^, **ZnO**^**1RR**^, and **ZnO**^**1T*****rac***^(b); **ZnO**^**2R**^, **ZnO**^**2S**^, and **ZnO**^**2*****rac***^ (c); and **ZnO**^**3R**^, **ZnO**^**3S**^, and **ZnO**^**3*****rac***^ (d). Each spectrum is averaged from 40 origin spectra.

Analysis of the spectra recorded for **ZnO**^**1RS**^ and **ZnO**^**1SR**^ capped with homochiral *cis*-aminoindanolates demonstrates that the patterns in the low wavenumber region, due to the homochiral shell and ZnO QD interaction, are essentially featureless (Figure 3a and Figures S21a and S22a) whereas for **ZnO**^**1C*****rac***^ with the heterochiral shell, two overlapping bands at 408 and 437 cm^–1^ are present (Figure 3a and Figure S22a). These bands are attributed to E_1_(TO) and E_2H_ ZnO modes. Remarkably, differences in the band intensities for *trans*-aminoindanolate-coated **ZnO**^**1RR**^ and **ZnO**^**1SS**^ are observed (Figure 3b and Figure S22b). The spectrum of **ZnO**^**1RR**^ is generally featureless with a few bands of very low intensities (slightly above the noise level), while for **ZnO**^**1SS**^, relatively sharp bands of low intensity at 420, 452, and 497 cm^–1^ are detected and assigned as E_2_ of ZnO (the first band) while two others are due to ligand vibration. For **ZnO**^**1T*****rac***^, only one weak broad band at 417 cm^–1^ is present (Figure 3b). Due to the broad shape of this band and its small intensity, the assignment of this band is not straightforward and, therefore, cannot be unambiguously recognized as vibration coming from the ligand or ZnO core. In contrast, in the case of **ZnO**^**2**^ and **ZnO**^**3**^ families, due to the spectral window for the respective proligands (Figures S21c,d and S22c,d), the observation of ZnO modes and their assignment is essentially simpler. For the aminophenylethanolate-coated **ZnO**^**2**^, in the low-wavenumber region, the band intensity is increasing, resulting in the appearance of bands characteristic for ZnO QDs with the hexagonal wurtzite structure (Figure 3c). Moreover, the intensity of these bands is strong enough to be observed in the Raman spectra recorded for **ZnO**^**2R**^, **ZnO**^**2S**^, and **ZnO**^**2*rac***^. Thus, bands at 328, 379, 399, and 433 cm^–1^ are recognized as the respective E_2H_-E_2L_, A_1_ (TO), E_1_(TO), and E_2H_ ZnO modes. What should be highlighted is the increased intensity of the E_1_(TO) mode at 399 cm^–1^ with respect to the intensities of three other modes. This is most probably due to the interaction with the ligand vibrations, especially in the case of **ZnO**^**2S**^ (Figure S22c). Remarkably, for the diphenylpyrrolidinemethanolate-coated **ZnO**^**3**^ (Figure 3d), all observed bands for this region are due to the ZnO vibration, as no Raman bands are observed in the spectrum of the respective proligands (Figures S21d and S22d). Thus, bands at 328, 381, 397, and 434 cm^–1^ are related to E_2H_–E_2L_, A_1_(TO), E_1_(TO), and E_2H_ ZnO modes (Figure 3d).

The second highlighted spectral region (950–1150 cm^–1^, Figure 3) is mostly dominated by the coating ligand vibrations; however, the observed bands are broadened due to the core–ligand interactions (Figures S21 and S23). In this area, ZnO combination modes and overtones are also present. Nevertheless, the assignment of these modes is complicated due to the bands overlapping; particularly, the high-intensity ligand bands often cause a screening effect of usually less intense ZnO combination modes. The spectra of **ZnO**^**1C**^ and **ZnO**^**1T**^ in this region are less informative, but the patterns recorded for **ZnO**^**2**^ and **ZnO**^**3**^ allow for deconvolution of the Raman bands and their detailed assignment (Figure S24). Thus, the bands at 968 and 997 cm^–1^ are typical for ZnO modes and are assigned to A_1_(LO) overtones and those at 1089 and 1157 cm^–1^ as E_1_ + A_1_ and E_2_ + A_1_ combination modes (Figure 3c,d and Figure S24). Further spectral analysis of **ZnO**^**1C**^ and **ZnO**^**1T**^ shows mutual changes of intensities of the two most remarkable bands above 1000 cm^–1^ (Figure 3a,b). Generally, for the heterochiral *cis*- and *trans*-aminoindanolate-capped QDs, the band at 1043 cm^–1^ dominates over the band at 1024 cm^–1^. The band at 1043 cm^–1^ is recognized as out-of-plane CH bending and CC stretching ligand vibrations; the band at 1024 cm^–1^ to the out-of-plane CH bending and mixed rocking vibrations of CH, NH_2_, and Zn–O_alkoxide_.

In the spectra of **ZnO**^**1SR**^ and **ZnO**^**1SS**^ with homochiral 1*S*,2*R*- and 1*S*,2*S*-aminoindanolates, respectively, the bands at 1033 or 1022 cm^–1^ are dominating over the second band at a higher wavenumber (1050 or 1041 cm^–1^). However, in the case of **ZnO**^**1RS**^ and **ZnO**^**1RR**^ with the homochiral 1*R*,2*S*- and 1*R*,2*R*-aminoindanolate, respectively, two bands have comparable intensities (1032 and 1050 cm^–1^ or 1025 and 1045 cm^–1^, respectively). A different situation is observed in the spectra of **ZnO**^**2**^ and **ZnO**^**3**^ families. For the heterochiral ligand-capped **ZnO**^**2*****rac***^ and **ZnO**^**3*****rac***^, the bands around 1000 cm^–1^ (which are attributed to the ligand skeletal mixing modes of out-of-plane bending CH and CCC) dominate over two other weak bands (1025 and 1072 cm^–1^ or 1035 and 1076 cm^–1^, respectively). The bands at 1072 and 1076 cm^–1^ are recognized as out-of-plane bending CH and stretching CO and in-plane bending CC ligand vibrations. At the same time, for the homochiral ligand-coated QDs (**ZnO**^**2R**^, **ZnO**^**2S**^ and **ZnO**^**3R**^, **ZnO**^**3S**^), the intensities of the respective bands around 1000 cm^–1^ are remarkably reduced (Figure 3c,d). Interestingly, in the case of the spectra recorded for **ZnO**^**2R**^ and **ZnO**^**2S**^ (Figure 3c), two new bands at 968 cm^–1^ (attributed to the CC stretching of the five-membered ring and the out-of-plane NH and CCN bending vibrations) and at 1053 cm^–1^ (ZnO overtones) are detected. In turn, for **ZnO**^**3R**^ and **ZnO**^**3S**^, increasing intensity and broadening of bands at 1079 or 1089 cm^–1^ are visible (Figure 3d). The tentative assignments of all vibrations of QDs observed in the Raman spectra are shown in Table S2.

The above analysis clearly demonstrates that it is very difficult to make definite recognition and identification between QDs coated with different chiral ligands based only on the differences of the respective Raman bands’ position shifts and their intensities. Generally, the characteristics of the obtained spectra are influenced by appearance of modes related to the inorganic core, the core–ligand interactions, and ligand vibrations. Therefore, an additional method is desired to enhance the Raman fingerprint recognition and get a more in-depth understanding of the vibrational spectra in relation to the chirality of the studied QDs. To tackle this challenge, PCA was applied.

### 2.3 PCA of the Raman Data

PCA transforms a large number of original correlated variables (Raman data) into a smaller number of uncorrelated variables called PCs. PCA can be described as uncorrelated linear combination of the original variables (*X*) as *X* = *t*^1^*p*′_1_ + *t*^2^*p*′_2_+ ... + *t*^A^*p*′_A_ + *E* = TP + *E*, where *A* is the total number of extracted PCs, *t* (scores) and *p* (loadings) are the new latent variables, and *E* is the residual matrix. The scores show how the studied data are related to each other while the loadings reveal the importance of the original variables for the patterns seen in the scores. Thus, we examined the calculated scores and loadings for the most important PCs, as determined from percent variance plots, and used them to investigate changes in the spectral features of the Raman data and to indicate the most important variables (fingerprints) and regions related to the differences or similarities found in the Raman data set.

**Figure 4:**
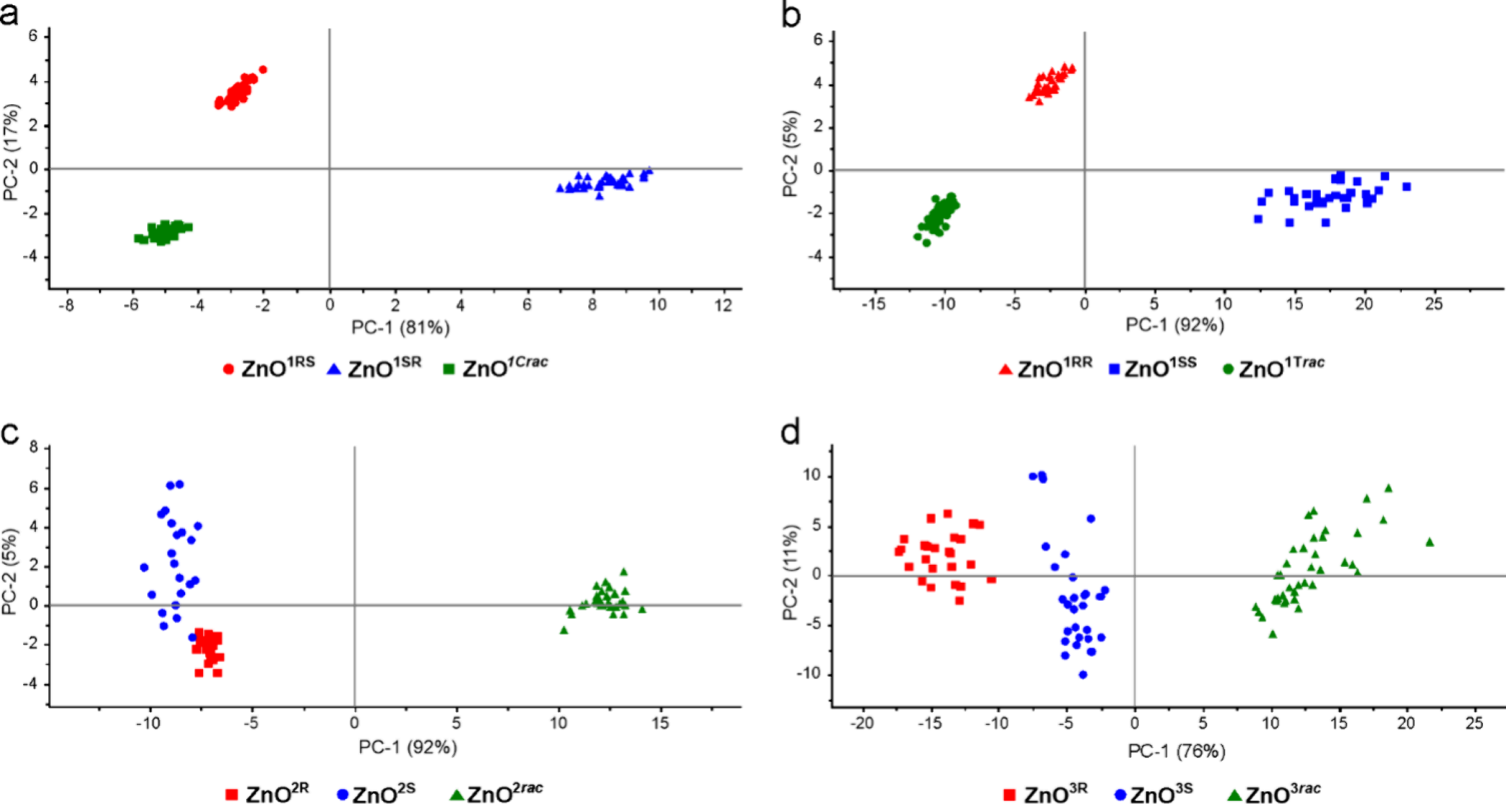
PCA scores of **ZnO**^**1SR**^, **ZnO**^**1RS**^, and **ZnO**^**1C*****rac***^ (a); **ZnO**^**1SS**^, **ZnO**^**1RR**^, and **ZnO**^**1T*****rac***^ (b); **ZnO**^**2R**^, **ZnO**^**2S**^, and **ZnO**^**2*****rac***^ (c); and **ZnO**^**3R**^, **ZnO**^**3S**^, and **ZnO**^**3*****rac***^ (d) for the 960–1120 cm^–1^ region.

Initially, PCA calculations performed for the whole recorded spectral region (200–2000 cm^–1^) show that the first and second principal components (PC1 + PC2) carry 98, 94, 89, and 76% of the variation among *cis*- and *trans*-aminoindanolate-, aminophenylethanolate-, and diphenylpyrrolidinemethanolate-capped QDs (Figure S24). Then, the PCA performed for the data in the 960–1120 cm^–1^ spectral region (Figure 4) revealed that the first and the second principal components (PC1, PC2) are the most significant and explain 98, 97, 97, and 87% of the variance in the data of **ZnO**^**1C**^, **ZnO**^**1T**^, **ZnO**^**2**^, and **ZnO**^**3**^ families. The sums of PC1 and PC2 values for all QDs families are shown in Table S3. In the case of **ZnO**^**1C**^ and **ZnO**^**1T**^, the scores calculated for homochiral ligand-capped **ZnO**^**1RS**^ and **ZnO**^**1SR**^ or **ZnO**^**1SS**^ and **ZnO**^**1RR**^ are separated from the scores of heterochiral ligand-capped **ZnO**^**1C*****rac***^ and **ZnO**^**1T*****rac***^ by the PC1 and PC2 axes (Figure 4a and b, respectively). Moreover, the scores of **ZnO**^**1RS**^ and **ZnO**^**1SR**^ as well as **ZnO**^**1SS**^ and **ZnO**^**1RR**^ are separated by the PC2 axis. For **ZnO**^**2**^ and **ZnO**^**3**^ families, the PC1 axis divided the calculated scores into two groups characteristic for QDs featuring the homochiral shell (**ZnO**^**2S**^, **ZnO**^**2R**^ and **ZnO**^**3S**^, **ZnO**^**3R**^) and the heterochiral shell (**ZnO**^**2*****rac***^, **ZnO**^**3*****rac***^; Figure 4c,d). Regarding the data obtained for **ZnO**^**2**^ presented in Figure 4c, it seems that **ZnO**^**2S**^ (blue) scores slightly cover **ZnO**^**2R**^ (red) scores. However, in 3D plot projection (Figure S25), all scores presented for the **ZnO**^**2**^ family are nicely separated. Notably, the calculated scores for heterochiral ligand-capped **ZnO**^**2*****rac***^ and **ZnO**^**3*****rac***^ are located in close proximity to the PC2 axis, but at the same time on the positive side of the PC1 axis, while the scores of homochiral ligand-capped **ZnO**^**2**^ and **ZnO**^**3**^ are on the negative side of the PC1 axis. It should be mentioned that with the smaller differences between the spectra, the respective scores are closer to each other.

**Figure 5:**
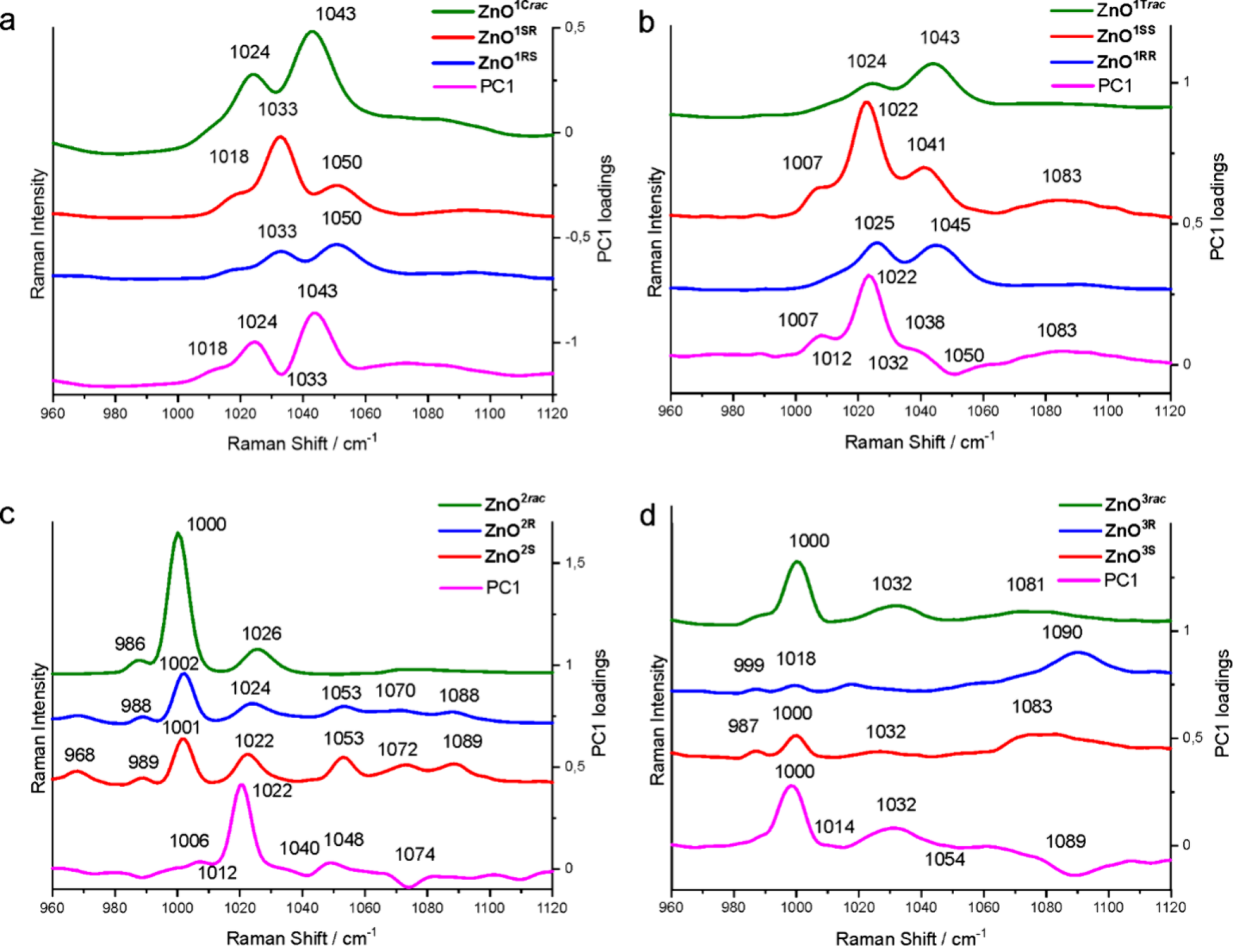
Raman spectra and PC1 loadings for **ZnO**^**1SR**^, **ZnO**^**1RS**^, and **ZnO**^**1C*****rac***^ (a); **ZnO**^**1SS**^, **ZnO**^**1RR**^, and **ZnO**^**1T*****rac***^ (b); **ZnO**^**2R**^, **ZnO**^**2S**^, and **ZnO**^**2*****rac***^ (c); and **ZnO**^**3R**^, **ZnO**^**3S**^, and **ZnO**^**3*****rac***^ (d).

For all studied QDs, each group of scores is significantly separated, and the differentiation is straightforward and thus can be limited to the first and second PCs. The data proves that a combination of Raman spectroscopy and PCA can be successfully used for the qualitative assay of chiroptically active QDs. Further analysis concerning the loadings of obtained PCs was performed in order to provide information on the intermolecular interactions based on the loading contributions (weightings) onto individual PCs and the variables (wavenumber of the spectrum) that are important for differentiation. Such an approach may reveal the variables corresponding to the most variability among the bands observed in the Raman spectra and stress the importance of the given molecular interactions observed as Raman bands indicating the fingerprints. Figure 5 shows the Raman spectra of studied QDs together with the PC1 loadings. Additionally, Table S3 presents the weighted variables in the loading of PC1. For **ZnO**^**1SR**^, **ZnO**^**1SR**^, and **ZnO^1C^***^**rac**^,* the variable at 1043 cm^–1^ has the largest weights (Figure 5a). Moreover, two other bands at 1024 and 1018 cm^–1^ have a small contribution to PC1 in the same direction as the most intensive one, while bands at 1033 and 1057 cm^–1^, both in the opposite direction, have much smaller influences on the PC1 value. The PC1 loadings of **ZnO**^**1SS**^, **ZnO**^**1RR**^, and **ZnO**^**1T*****rac***^ show that the variable at 1022 cm^–1^ has the largest weight (Figure 5b). The variables at 1007, 1038, and 1083 cm^–1^ have a small contribution to PC1 in the same direction as the variable at 1022 cm^–1^, while bands at 1012, 1032, and 1050 cm^–1^ have a weight in the opposite direction. Taking into account the weight of each of these PC1 loadings (Table S3), it is obvious that within aminoindolate-capped QDs, there are two main bands in the Raman spectra that can work as fingerprints, i.e., the band at 1043 cm^–1^ for **ZnO**^**1C**^ and the band at 1022 cm^–1^ for **ZnO**^**1T**^. For the **ZnO^2^** family the variable at 1022 cm^–1^ and for the **ZnO**^**3**^ family at 1000 cm^–1^ have the largest weights on the calculated PC1 values (Figure 5c,d).

**Figure 6:**
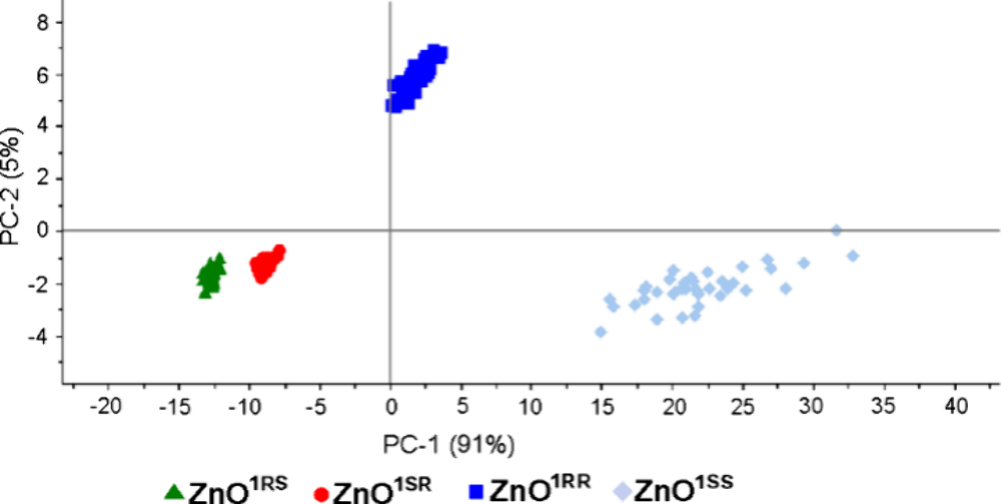
PCA scores of **ZnO**^**1RS**^, **ZnO**^**1SR**^, **ZnO**^**1RR**^, and **ZnO**^**1SS**^ for the 960–1120 cm^–1^ region.

In the next step, we wondered if the elaborated method of differentiation can be applied to differentiate between QDs capped with homochiral *cis*- and *trans*-1-amino-2-indanolate ligands, i.e., **ZnO**^**1RS**^, **ZnO**^**1SR**^, **ZnO**^**1RR**^, and **ZnO**^**1SS**^. With this aim, the PCA calculations were performed based on the Raman spectra recorded in the whole 200–2000 cm^–1^ region (Figure S26) and for the 960–1120 cm^–1^ region, including the most prominent bands (Figure 6). The calculated sum of PC1 and PC2 values for the reduced region explained 96% of the total variance among the studied samples (Table S3). The calculated scores for **ZnO**^**1RS**^ and **ZnO**^**1SR**^, both coated with *cis*-aminoindanolate ligands, are on the negative side of the PC1 axis, while the scores of both *trans*-isomer-coated **ZnO**^**1RR**^ and **ZnO**^**1SS**^ are gathered on the positive side of the PC1 axis. Interestingly, the calculated scores of **ZnO**^**1RS**^ and **ZnO**^**1SR**^ are gathered in two groups on the negative side of the PC1 and PC2 axes. Meanwhile, it is possible to make a differentiation between **ZnO**^**1RR**^ and **ZnO**^**1SS**^ based on the PC2 axis. Thus, the data strongly indicate the possibility of simultaneous classification among four ZnO QDs capped with different forms of chiral aminoindanolate ligands. Furthermore, to identify bands that are the most significant for differentiation purposes, the loadings of the first principal component (PC1) were plotted against the Raman data, indicating the 1022 cm^–1^ variable as the most important, and with the highest and positive weights on the finally calculated value of PC1 (Figure S27). Furthermore, the PC1 value is also influenced by three other lower-weighted bands at 1006, 1042, and 1082 cm^–1^. Variables with the opposite direction at 1012 and 1033 cm^–1^ are weighted almost zero (Table S3).

**Figure 7:**
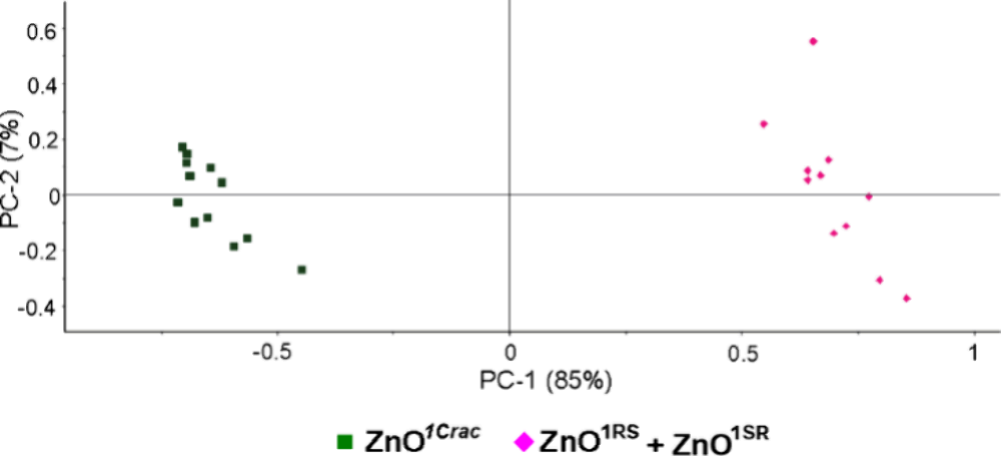
PCA scores of **ZnO**^**1*****rac***^ and a mixture of **ZnO**^**1RS**^ and **ZnO**^**1SR**^ for the 960–1120 cm^–1^ region.

Finally, a control experiment was performed to check if it is possible to use PCA to differentiate between QDs with a heterochiral ligand shell (i.e., QDs coated with a racemic mixture of enantiomers) and the respective mixture of homochiral ligand-coated QDs. For this purpose, a solid–solid mixture of **ZnO**^**1RS**^ and **ZnO**^**1SR**^ in the 1:1 ratio was prepared by mechanical mixing. All bands observed in the Raman spectrum of the **ZnO**^**1RS**^ and **ZnO**^**1SR**^ mixture are comparable to that observed for **ZnO**^**1C*****rac***^ as well as to that gathered independently for **ZnO**^**1RS**^ and **ZnO**^**1SR**^ (Figure S28). Nevertheless, PCA calculations based on the Raman spectra for the 200–2000 cm^–1^ (Figure S29) and 960–1120 cm^–1^ (Figure 7) regions enable to differentiate efficiently between **ZnO**^**1C*****rac***^ and a mixture of **ZnO**^**1RS**^ and **ZnO**^**1SR**^. The scores of **ZnO**^**1C*****rac***^ and the scores of the **ZnO**^**1RS**^ and **ZnO**^**1SR**^ mixture are separated by the PC1 axis. The calculated sum of PC1 and PC2 values for the 960–1120 cm^–1^ region explained 92% of the total variance among the studied samples.

## 3 Conclusions

Characterization of NCs with ligand shells composed of mixed ligands remains a particularly great challenge, and interfacing QDs with optically active and racemic ligand systems introduces another level of entanglement. In this report, we combined an experimental analysis and PCA of the Raman spectra of ZnO QDs coated by structurally diverse homo- and heterochiral aminoalcoholate ligands. PCA significantly mitigates the Raman band analysis to the most important variables (fingerprints), with the highest weighting on the differentiation among the samples. For example, the analysis indicated that bands dominated by aminoalcoholate ligand vibrations at 1043 cm^–1^ (**ZnO**^**1C**^), 1022 cm^–1^ (**ZnO**^**1T**^), 1022 cm^–1^ (**ZnO**^**2**^), and 1000 cm^–1^ (**ZnO**^**3**^) play the most important role for the differentiation purposes. The calculated values of PC1 + PC2 explain 98, 97, 97, and 87% (**ZnO**^**1C**^, **ZnO**^**1T**^, **ZnO**^**2**^, and **ZnO**^**3**^, respectively) variance among studied samples. Holistically, this work demonstrates that the presented high-throughput screening method is a powerful technique for the fingerprint identification and allows efficient differentiation between QDs with homochiral ligand shells, and also QDs coated with a racemic mixture of enantiomers.

## 4 Methods

### 4.1 Materials

Diethylzinc (ABCR) was used as solution in dry hexane. (1*S*)-2-Amino-1-phenylethanol (ABCR), (1*R*)-2-amino-1-phenylethanol (ABCR), (1*R*,2*S*)-*cis*-1-amino-2-indanolate (Aldrich), (1*S*,2*R*)-*cis*-1-amino-2-indanolate (Aldrich), (1*S*,2*S*)-*trans*-1-amino-2-indanolate (Aldrich), (1*R*,2*R*)-*trans*-1-amino-2-indanolate (Aldrich), (*R*)-α,α-diphenyl-2-pyrrolidinemethanol (Aldrich), and (*S*)-α,α-diphenyl-2-pyrrolidinemethanol (Aldrich).

### 4.2 Zinc Oxide QD Synthesis

ZnO QDs were prepared using a previously reported procedure.^61^ To a THF solution of selected aminoalcohol (1.0 mmol), diethylzinc in hexane (0.5 mL, 1.0 mmol) was added dropwise and stirred at ca. −40 °C for several minutes. Then, the reaction mixture was allowed to warm to room temperature, stirred for 2 h, and exposed to oxygen and water from air for 5 days. Hexane was added to separate ZnO QDs (except for **ZnO**^**3**^) from the parent THF solution. To remove excess aminoalcohol liberated during ZnO synthesis, **ZnO**^**1C**^, **ZnO**^**1T**^, and **ZnO**^**2**^ were dissolved in THF participated by hexane three times and **ZnO**^**3**^ was washed several times with THF.

### 4.3 Raman Spectroscopy Measurements

Raman spectroscopy measurements were carried out in the mapping mode (1 μm × 1 μm). The spectra were taken in different places of the sample using the Renishaw inVia Raman system equipped with the 785 nm diode laser. The light from the laser was passed through a line filter and focused on a sample mounted on an X–Y–Z translation stage with a 50× microscope objective, NA = 0.25. The beam diameter was approximately 2.5 μm. The laser power at the sample was 5 mW or less. Raman data were collected from three different batches (three samples for each type of QDs) in at least 10 different places.

### 4.4 PCA

PCA was performed over the preprocessed Raman spectra. First, Raman spectra were smoothed with a Savitsky–Golay filter, the background was removed using baseline correction (10 AQitenary and 64 points), and then the spectra were normalized using a so-called min–max normalization using a built-in OPUS software package (Bruker Optic GmbH 2012 version). After that, the data were transferred to the Unscrambler software (CAMO software AS, version 10.3, Norway), where the PCA calculation was performed based onto the NIPALS algorithm, validation (random with 20 segments), significance 0.05, and the 90 number of samples (Raman spectra).

## Supporting Information Available

Additional QD characterization data: TEM, CD, Raman, and PCA (PDF)
