# Supplementary material for: Direct Readout of Homo- vs Heterochiral Ligand Shell of Quantum Dots
Source: ACS Appl Mater Interfaces. 2024 Jul 8;16(28):37308–17. doi: 10.1021/acsami.4c07648 (PMC11261568; doi:10.1021/acsami.4c07648)
Supplement: Supplementary file 1 — am4c07648_si_001.pdf [file am4c07648_si_001.pdf]

Supporting Information

Direct Readout of Homo- vs. Heterochiral Ligand Shell of Quantum Dots

Elżbieta Chwojnowska,[†,§,*], *Aneta A. Kowalska,*[†,§,*] *Agnieszka Kamińska*[†] *and Janusz Lewiński*[†,‡,*]

[†]Institute of Physical Chemistry Polish Academy of Sciences, Kasprzaka 44/52, 01-224 Warsaw, Poland

[‡]Faculty of Chemistry,Warsaw University of Technology, Noakowskiego 3, 00-664Warsaw, Poland

§ Authors declare equal contribution

Corresponding authors: echwojnowska@ichf.edu.pl (EC), akowalska@ichf.edu.pl (AK), janusz.lewinski@pw.edu.pl (JL)

Table of Contents

Characterization of ZnO QDs **S2**

Transmission Electron Microscopy **S2**

Circular dichroism **S12**

Raman Spectroscopy **S14**

Principal component analysis **S19**

Characterization of ZnO QDs

Transmission Electron Microscopy

Size, shape and morphology of the nanoparticles were examined by High-Resolution Transmission Electron Microscopy (HR TEM). Nanoparticle samples were drop-cast (THF or DMSO solution) onto 300-mesh, holey carbon-coated copper grids (Quantifoil). Afterward, the excess solvent evaporated at room temperature. Nanoparticle samples were imaged using a C_s corrected scanning transmission electron microscope (STEM, HITACHI HD2700, 200 kV). The observations were carry on in three modes: SE (images used to study morphology), HAADF STEM (Z-contrast) and HR TEM (images showing the atomic structure). A wide variety of magnifications (from x1500 up to x8000000) were used to study the microstructure of ZnO samples. The size of nanoparticles was calculated by image analyses, using ImageJ2x computer software. For image analyses a population of 100 particles was used for each sample. Average sizes and standard deviations were calculated from obtained results.

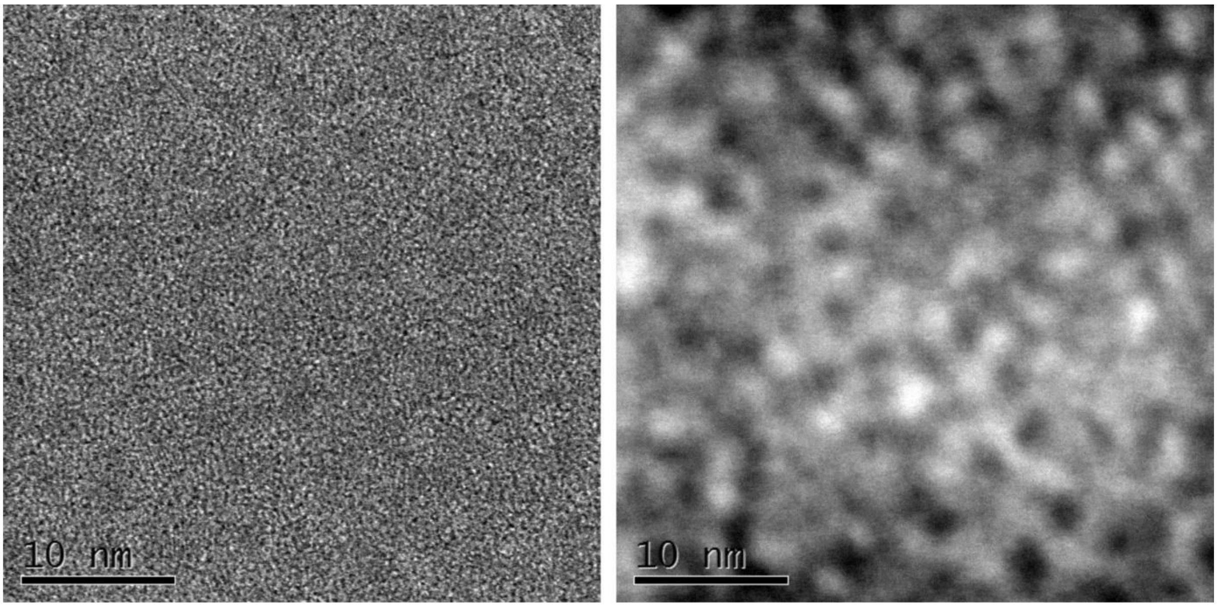

Figure S1. Representative HR TEM (left) and HAADF STEM (right) images of **ZnO^{1RR}** dispersed in THF.

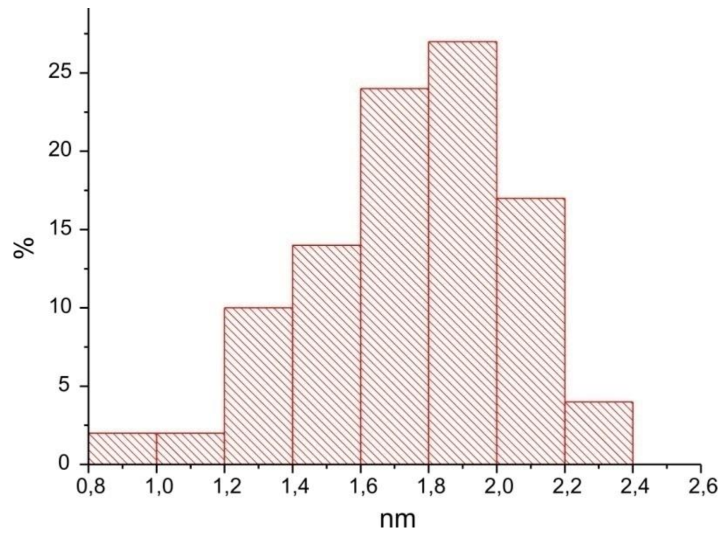

Figure S2. Size distribution of **ZnO^{1RR}** calculated from TEM images. Average size of the inorganic core: 1.7±0.3 nm.

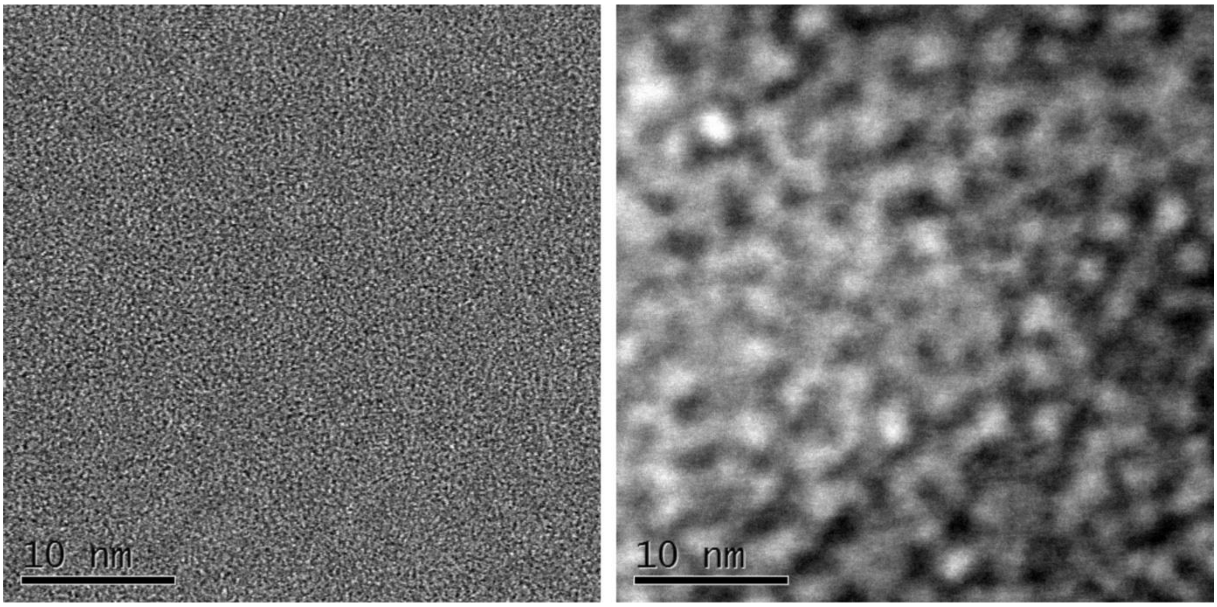

Figure S3. Representative HR TEM (left) and HAADF STEM (right) images of **ZnO^{1SS}** dispersed in THF.

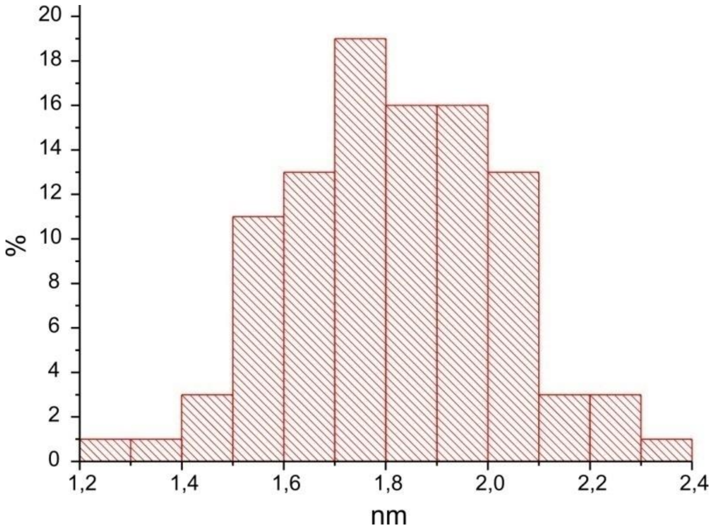

Figure S4. Size distribution of **ZnO[1SS]** calculated from TEM images. Average size of the inorganic core: 1.8±0.2 nm.

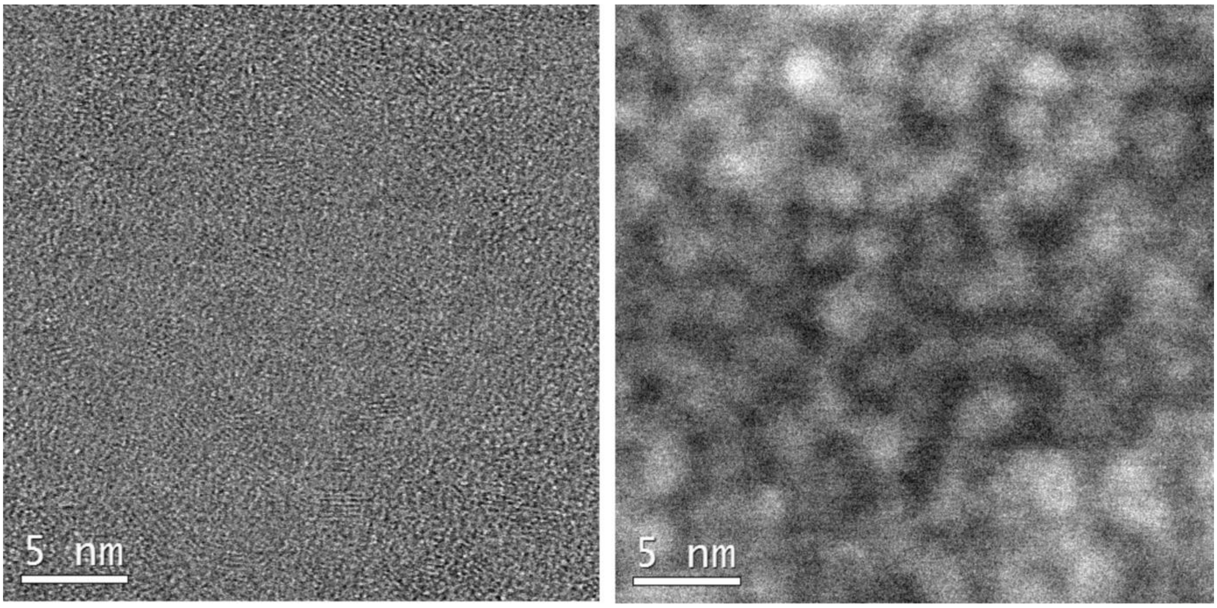

Figure S5. Representative HR TEM (left) and HAADF STEM (right) images of ***ZnO[1Trac]*** dispersed in THF.

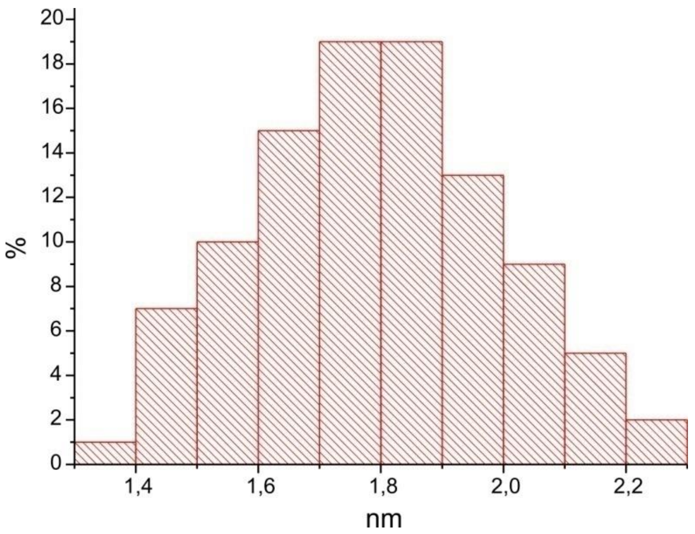

Figure S6. Size distribution of ***ZnO*** ^{1Trac} calculated from TEM images. Average size of the inorganic core: 1.8±0.2 nm.

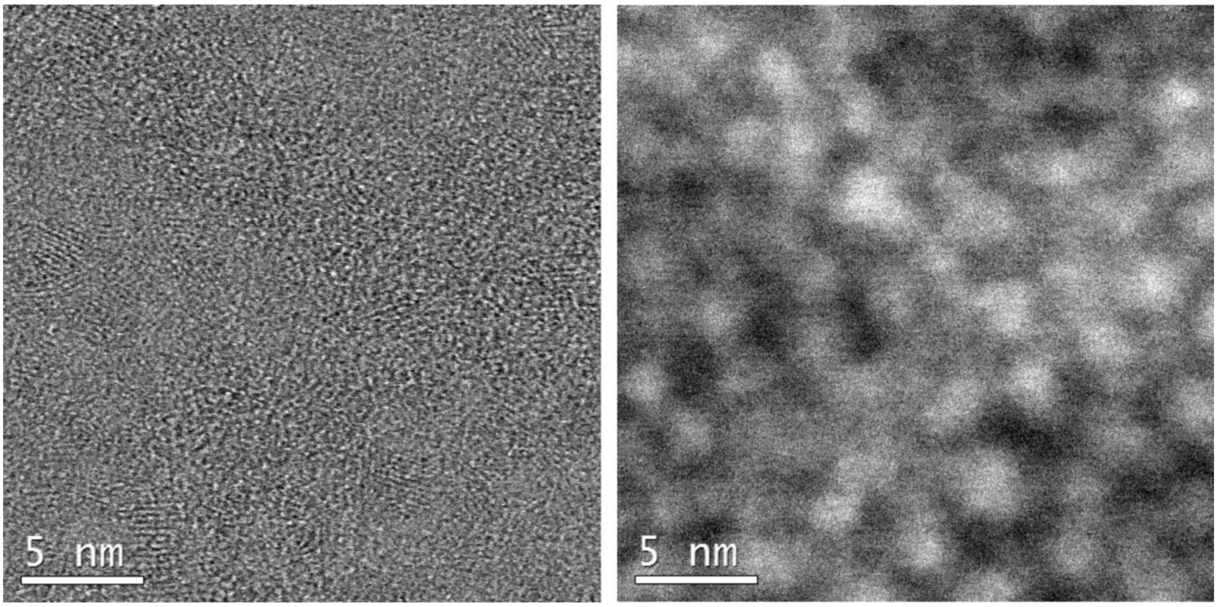

Figure S7. Representative HR TEM (left) and HAADF STEM (right) images of ***ZnO*** ^{1Crac} dispersed in THF.

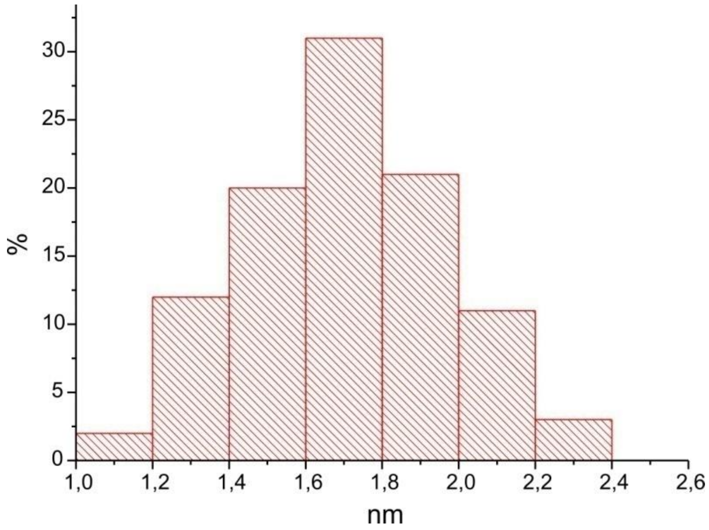

Figure S8. Size distribution of ***ZnO^{1Crac}*** calculated from TEM images. Average size of the inorganic core: 1.7±0.3 nm.

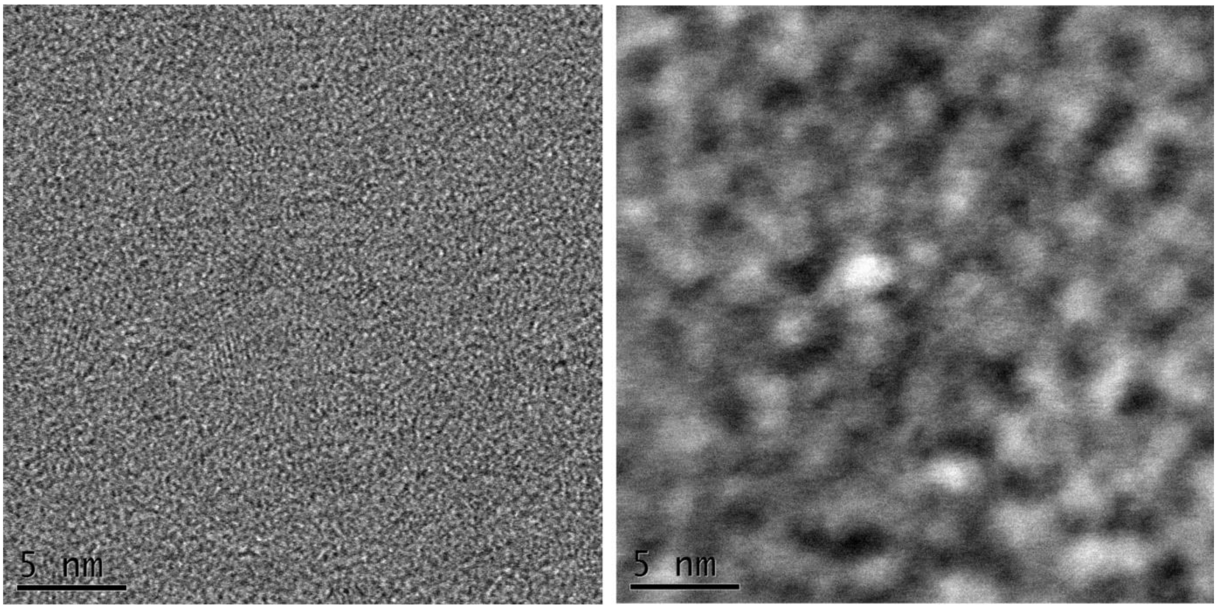

Figure S9. Representative HR TEM (left) and HAADF STEM (right) images of **ZnO^{1RS}** dispersed in THF.

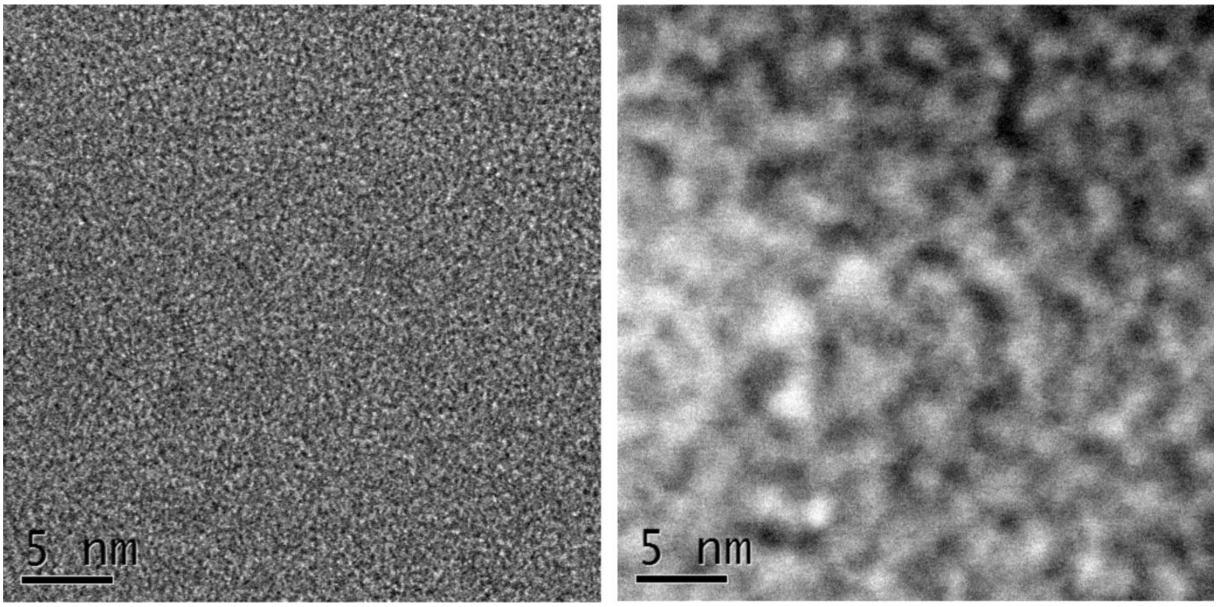

Figure S10. Representative HR TEM (left) and HAADF STEM (right) images of **ZnO**$^{\mathbf{1SR}}$ dispersed in THF.

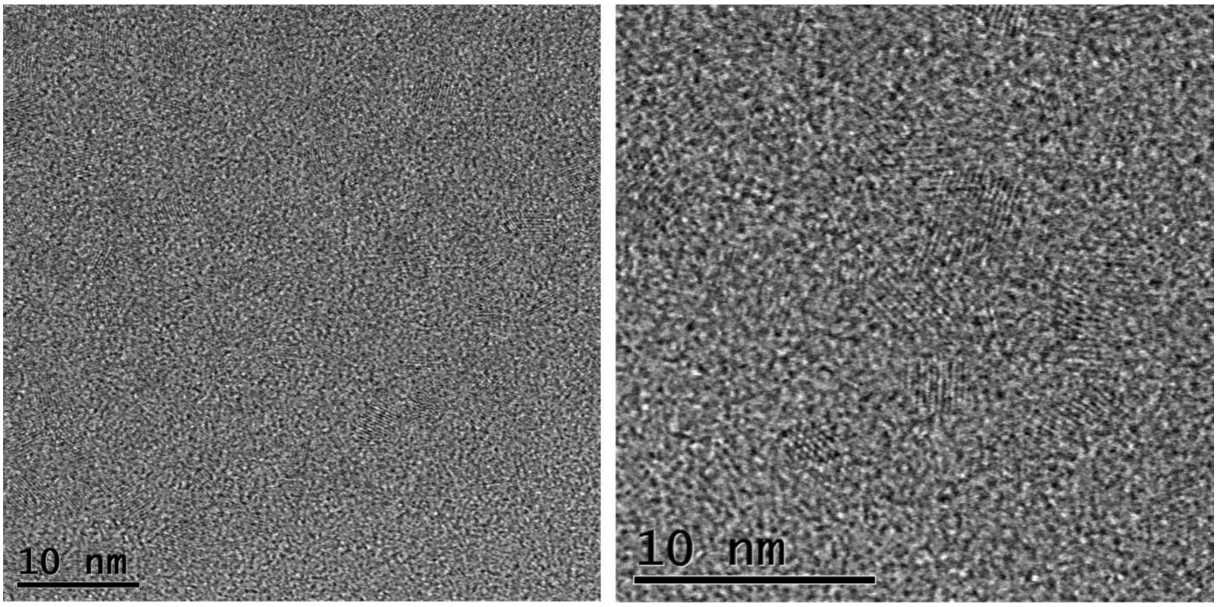

Figure S11. Representative HR TEM images of **ZnO**$^{\mathbf{2}\boldsymbol{rac}}$ dispersed in THF.

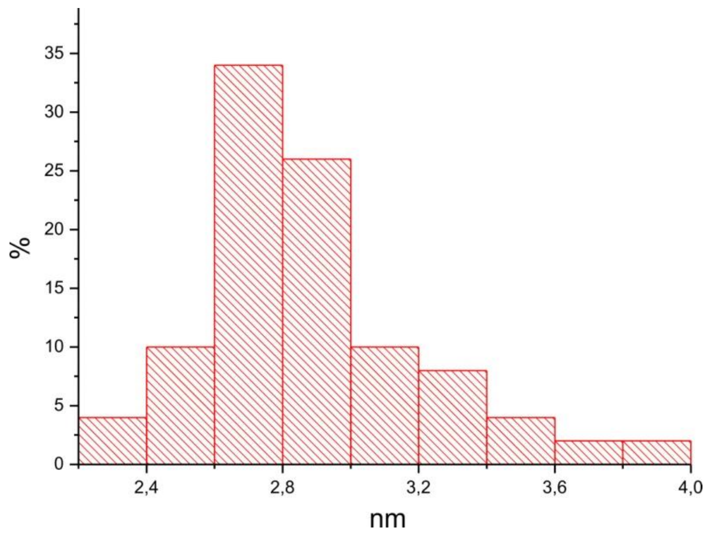

Figure S12. Size distribution of **ZnO^{2rac}** calculated from TEM images. Average size of the inorganic core: 2.9±0.4 nm.

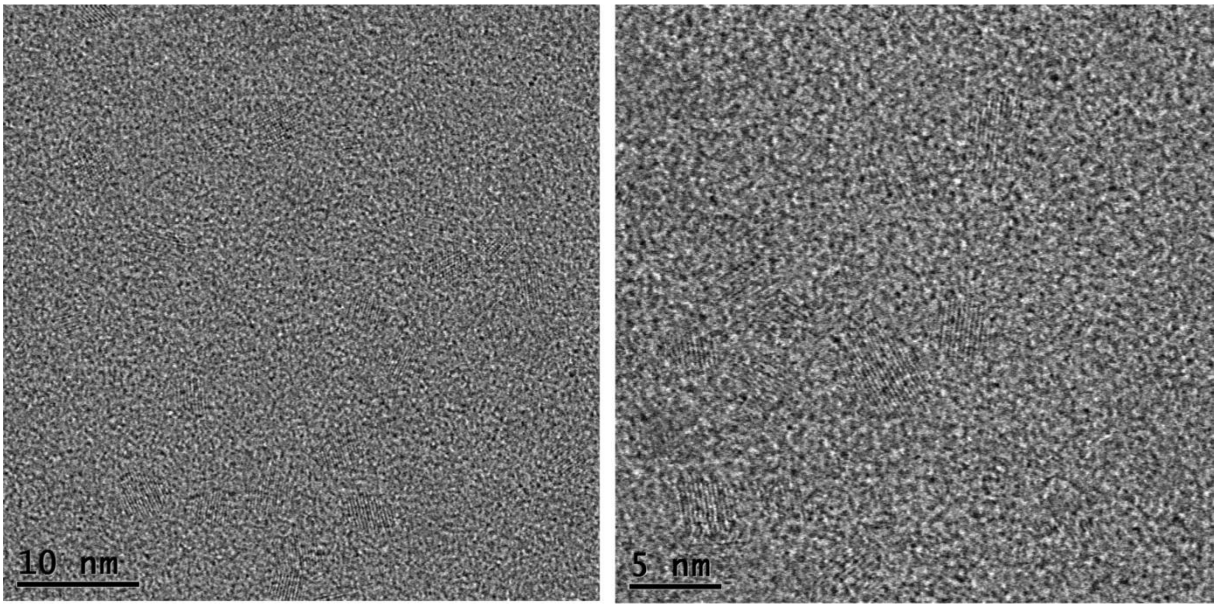

Figure S13. Representative HR TEM images of **ZnO^{2R}** dispersed in THF.

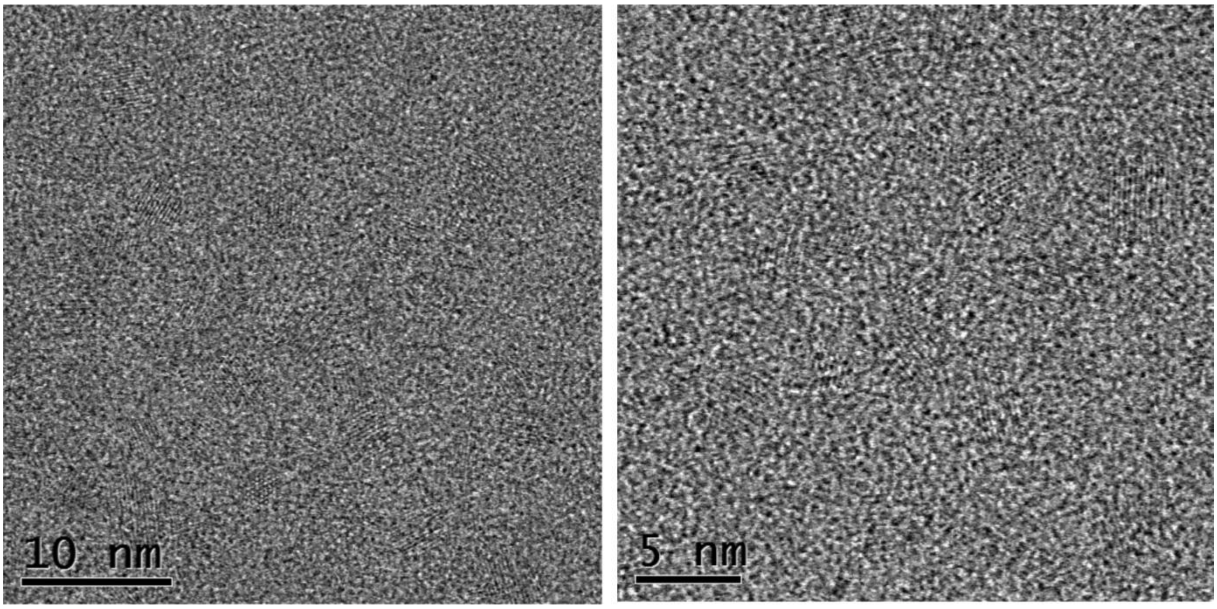

Figure S14. Representative HR TEM images of **ZnO**$^{\mathbf{2S}}$ dispersed in THF.

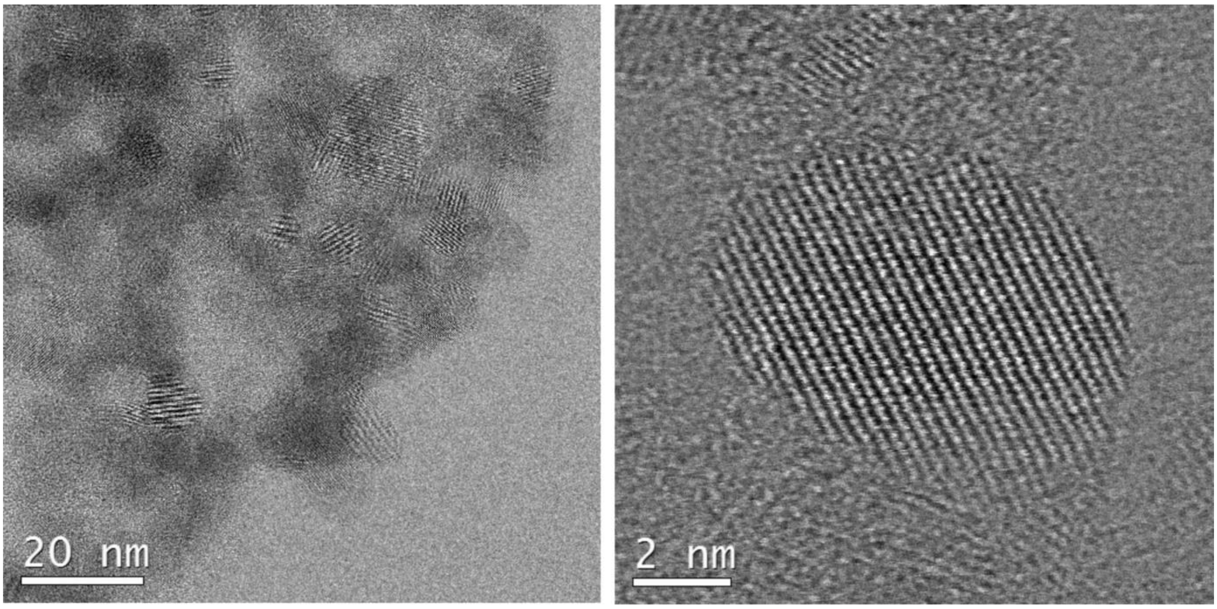

Figure S15. Representative HR TEM images of **ZnO**$^{\mathbf{3}\boldsymbol{rac}}$ dispersed in THF.

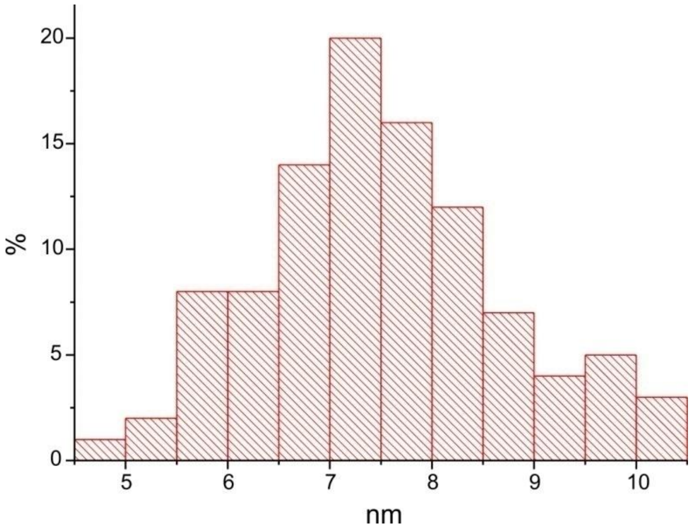

Figure S16. Size distribution of **ZnO**^{3rac} calculated from TEM images. Average size of the inorganic core: 7.5 ± 1,2 nm.

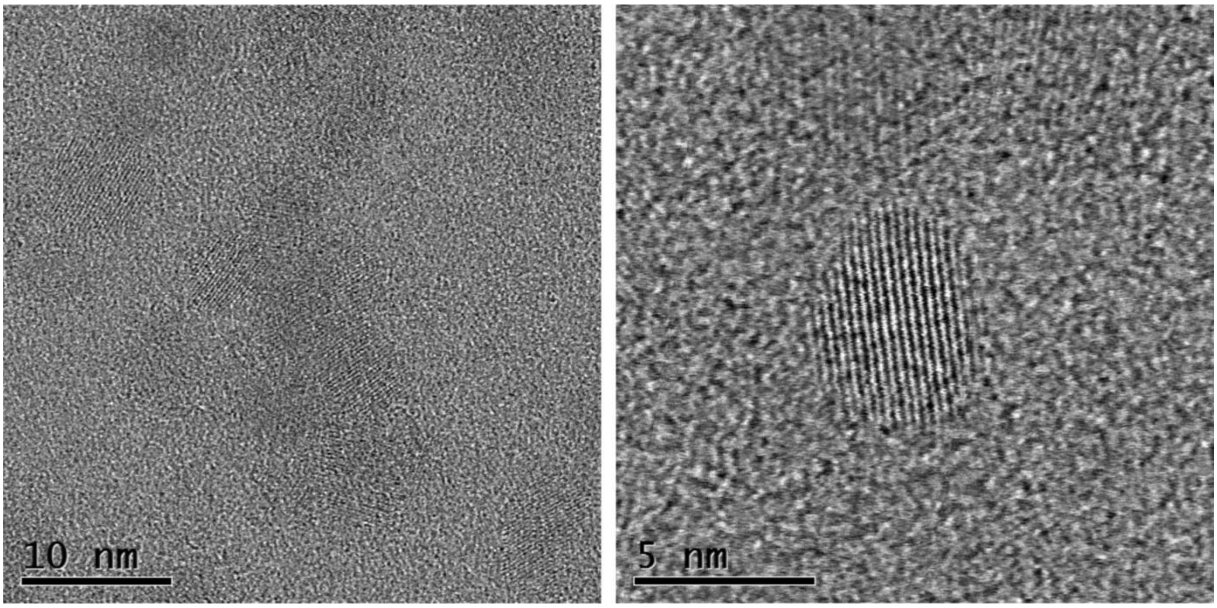

Figure S17. Representative HR TEM images of **ZnO**^{3R} dispersed in DMSO.

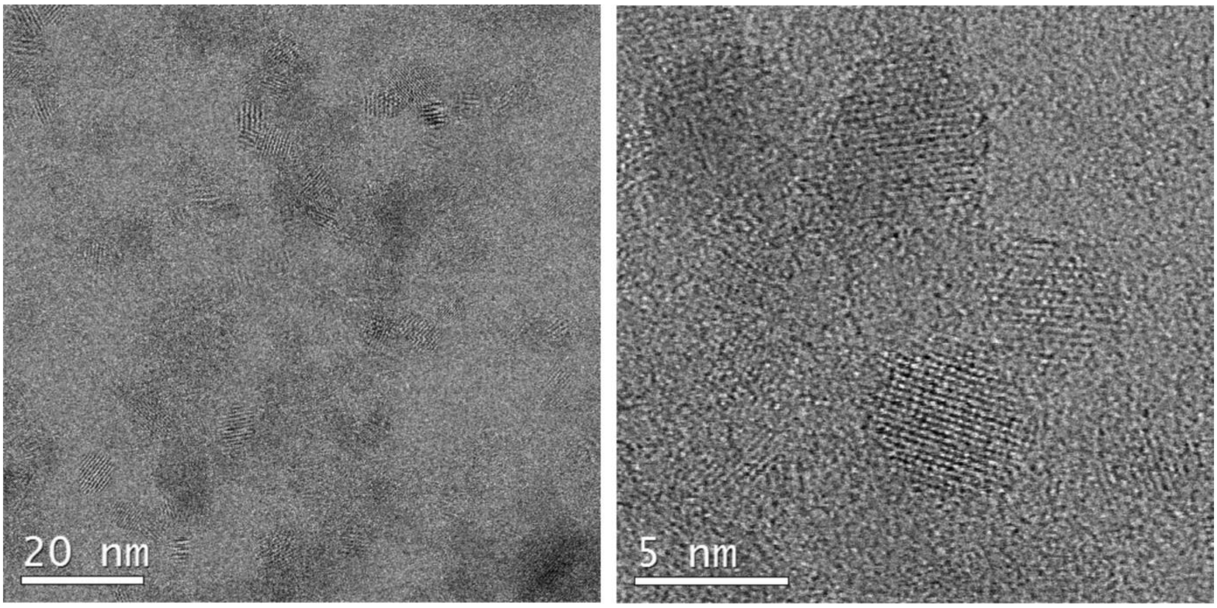

Figure S18. Representative HR TEM images of **ZnO^{3S}** dispersed in DMSO.

Average sizes of the inorganic core of **ZnO^{1RS}, ZnO^{1SR}, ZnO^{2R}**, **ZnO^{2S}**, **ZnO^{3R}** and **ZnO^{3S}** are consistent with previously reported.[1] (1.7 ± 0.2 nm, 1.7 ± 0.2 nm, 2.9 ± 0.4 nm, 2.9 ± 0.4 nm, 7.5 ± 1.4 nm, 7.5 ± 1.4 nm)

Circular dichroism

Circular dichroism (CD) spectra were recorded with a Jasco J-815 spectropolarimeter in THF or DMSO solution. Absorption analysis was carried out by using a Hitachi U-2910 spectrophotometer, with solvents as reference.

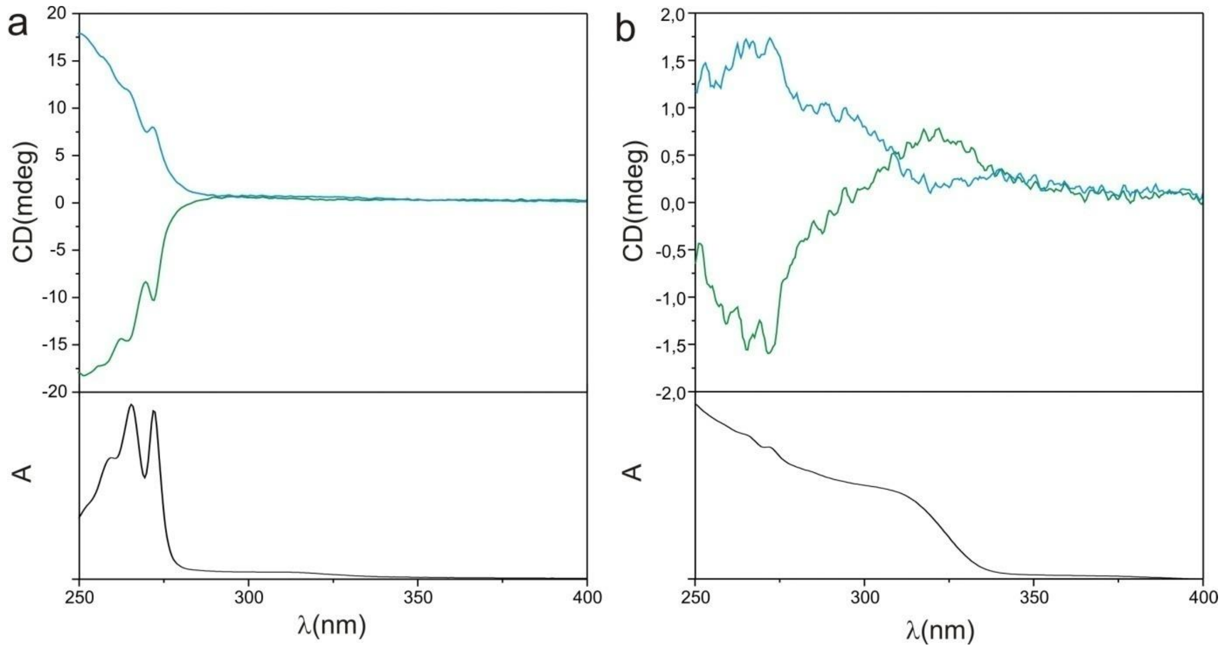

Figure S19. CD and normalized absorption spectra of proligands (a): **1RR** (green), **1SS** (blue); ZnO NCs (b): **ZnO^{1RR}** (green), **ZnO^{1SS}** (blue).

Absorption spectra of **ZnO^{1RR}** and **ZnO^{1SS}** are essentially identical and on Figure S19 only one representative spectrum is shown.

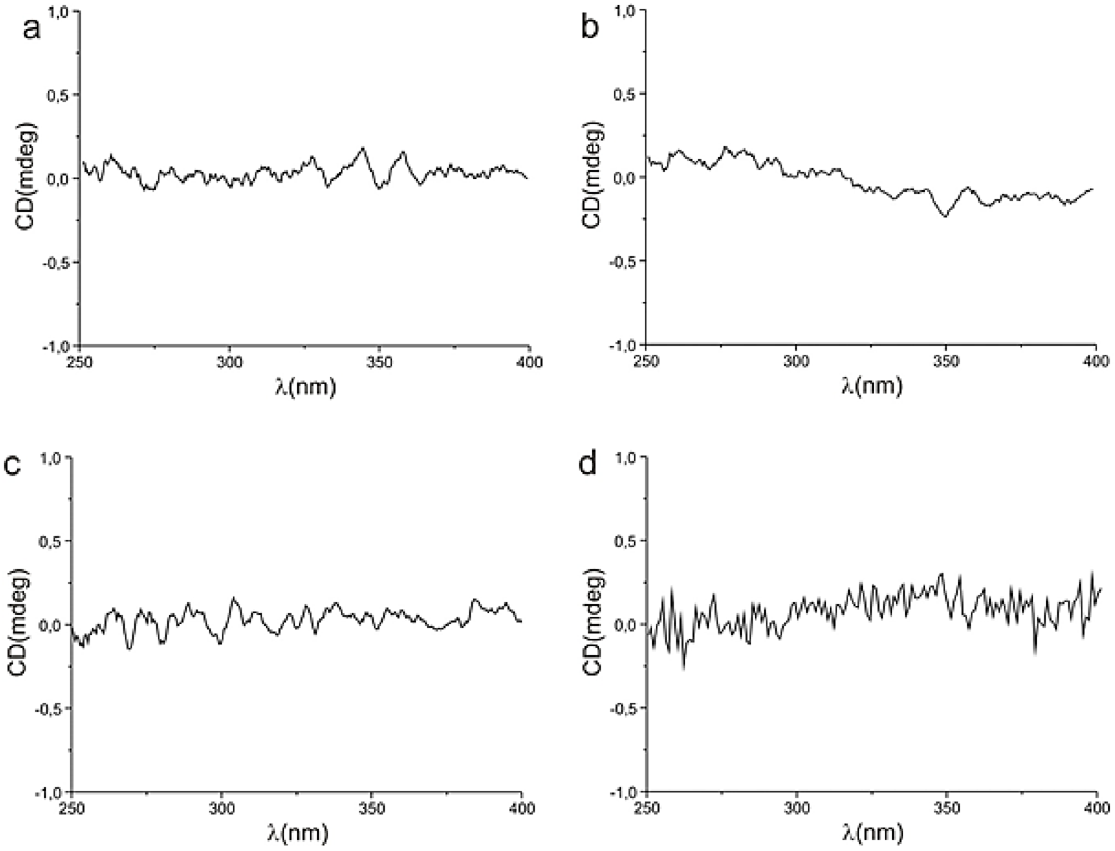

Figure S20. CD spectra of ZnO NCs: **ZnO$^{1C\mathit{rac}}$** (a); **ZnO$^{1T\mathit{rac}}$** (b); **ZnO$^{2\mathit{rac}}$** (c); **ZnO$^{3\mathit{rac}}$** (d).

CD spectra of **ZnO^{1RS}**, **ZnO^{1SR}**, **ZnO^{2S}**, **ZnO^{2R}**, **ZnO^{3S}** and **ZnO^{3R}** are consistent with previously reported. [1]

Raman Spectroscopy

Raman Spectroscopy measurements were carried out in the mapping mode (1mμ x 1mμ). The spectra were taken in different places of the sample (over 40) using the Renishaw inVia Raman system equipped with the 785 nm diode laser. The light from the laser was passed through a line filter and focused on a sample mounted on an X–Y–Z translation stage with a 50 × microscope objective, NA = 0.25. The beam diameter was approximately 2.5 μm. The laser power at the sample was 5 mW or less.

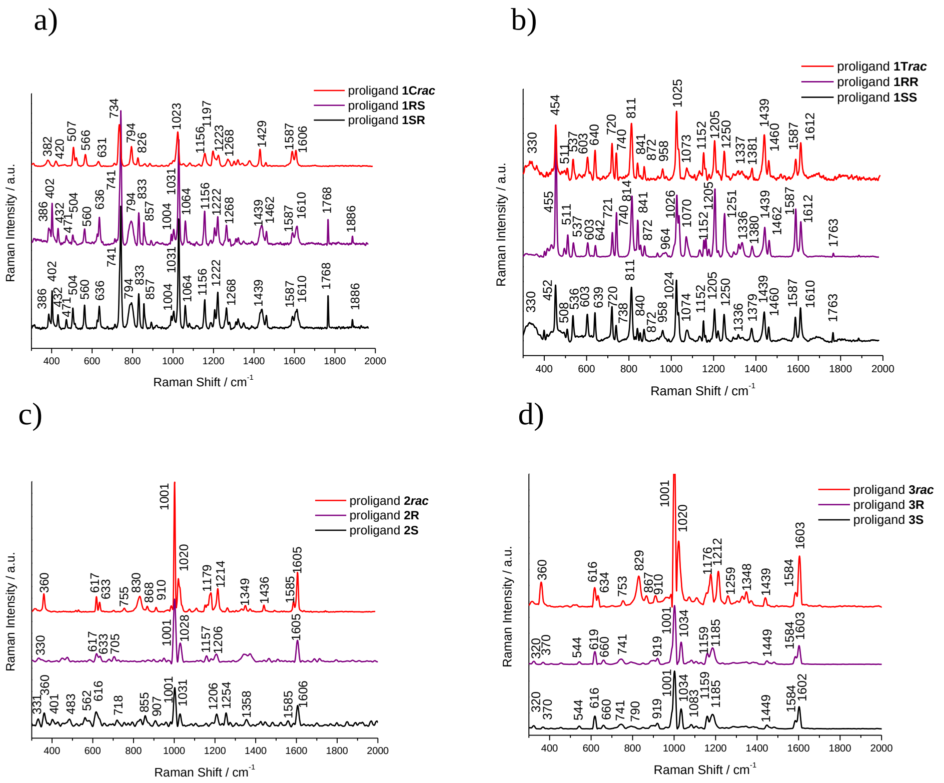

Figure S21. The Raman spectra of **1C*rac***, **1SR**, **1RS** (a); **1T*rac***, **1RR**, **1SS** (b); **2*rac***, **2S**, **2R** (c); **3rac, 3S, 3R** proligands (d).

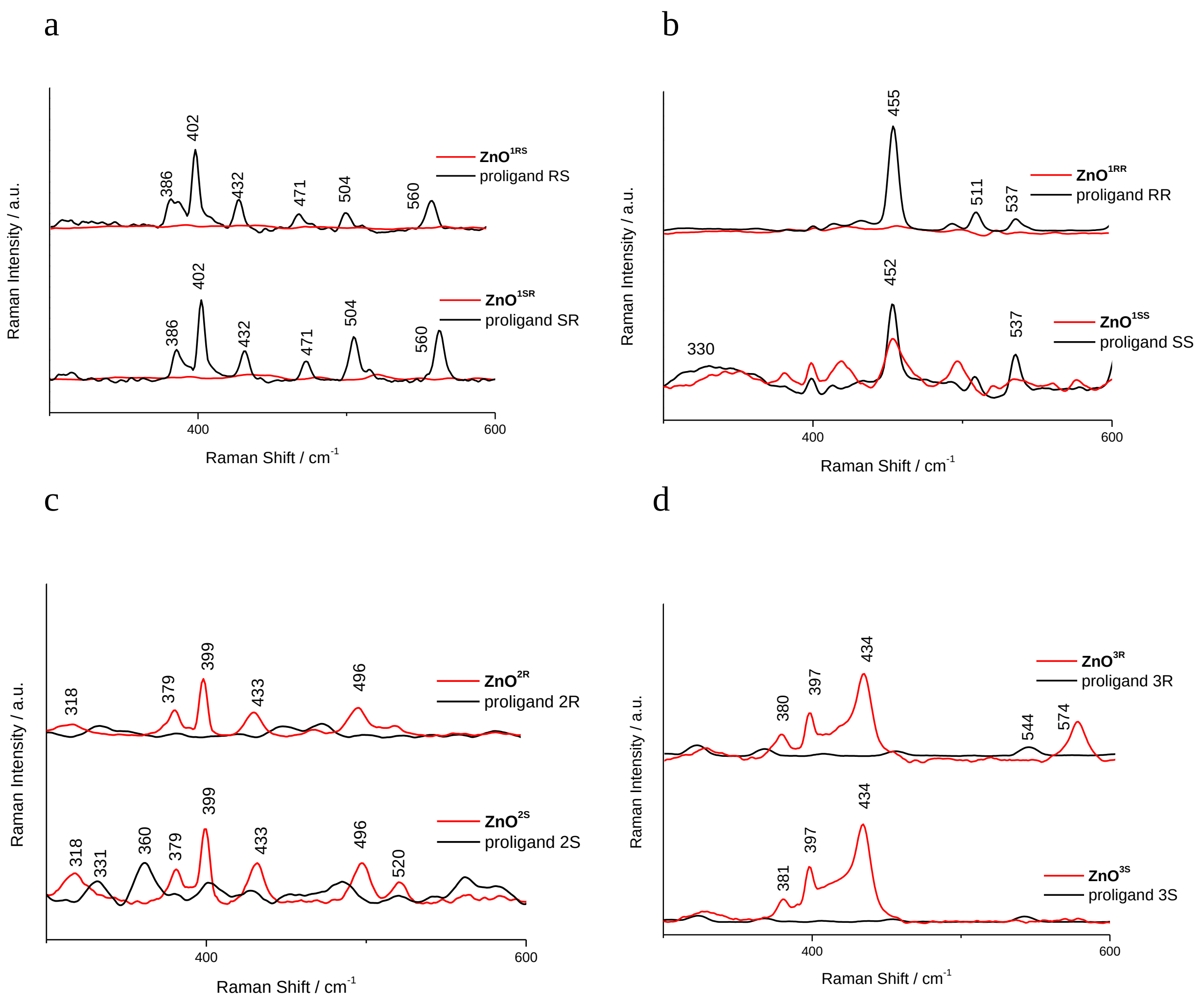

Figure S22. The comparison of the Raman spectra recorded for ZnO NCs and proligandsin the 300-600 cm^{-1}region: **ZnO^{1RS}**, **ZnO^{1RS}**, and **1RS**, **1SR** (a); **ZnO^{1RR}**, **ZnO^{1SS}**, and **1RR**, **1SS** (b); **ZnO^{2R}**, **ZnO^{2S}**, and **2S, 2R** (c); **ZnO^{3R}**, **ZnO^{3S}**, and **3S, 3R** (d).

Table S1. Tentative assignments of the Raman bands observed for proligands. [2,3,4]

Raman band positions [cm^{-1}]				Assignments*
**1SS / 1RR**	**1SR / 1RS**	**2S / 2R**	**3S / 3R**	
330/-		331/330	320	γ CCC + γ CH,
	386	360/-	370	δ CC + δ OH,
	402	401/-		δ CN + δ CCNH,
	432			δ CN + δ CCNH,
452 /455	471	483		νbenz. angle bend
508/511	504			

536/537	560	562	544	
603		616/617		CCC angle bend (5-mem.), ω CH, ω NH
639/642	636	-/633	616/619	
			660	
720/721		718/705		ν CC (5-mem.),
738/740	741		741	
-	794		790/-	ν CC (5-mem.), γ CH + γ COH
811/814				NH_2, γ NH + γ CCN, CH wag,
840/841	833			
872	857	855/-		
		907/-	919	CH_2 rock
958/964				ν CC (5-mem.), γ NH + γ CCN
	1004	1001	1001	γ CH + γ CCC,
1024/1026	1031	1031/1028	1034	ρ (OH) + (CH) + (NH2),
1074/1070	1064		1083/-	ν CO δ CC,
1152	1156	-/1157	1159	ν C=C + δ CH,
1178	1186	1186	1185	
1205	1206	1206	-	δ CH + δ COH
-	1222	-	-	δ CH + δ CCH
1250/1251	1268	1254/-		
1336		1358/1346		
1379/1380				δ NH + δ CH
1439	1439		1449	ν C=C + δ CH
1460/1462	1462			
1587	1587	1585	1584	ν C=C + δ CH
1610/1612	1610	1606/1605	1602/1603	ν C=C + ν CO

***ν: stretching, δ: in-plane bending, γ: out-of-plane bending, ω: wagging, ρ: rocking, comb. mod.: combination modes.**

Table S2. Tentative assignments of the Raman bands observed in the spectra of chiral ZnO NCs. [2, 3, 4]

Raman band positions [cm^{-1}]				Assignments*
**ZnO^{1SS} / ZnO^{1RR}**	**ZnO^{1SR} / ZnO^{1RS}**	**ZnO^{2S} / ZnO^{2R}**	**ZnO^{3S} / ZnO^{3R}**	
-	-	328/328	328/328	γ CCC + γ CH, ZnO (E_{2H}-E_{2L})
-	-	379/379	380/381	δ CC + δ OH, ZnO (A_1(TO))
-	-	399/399	397/397	δ CN + δ CCNH, ZnO (E_2)
420	-	433/433	434/434	δ CN + δ CCNH, ZnO (E_{2H})
452/459	-	-	-	νbenz. angle bend, ZnO comb. mod.
497/-	-	-	-	
649, 679, 720/728	689/692	-	-	CCC angle bend (5-mem.), ω CH, ω NH
-	753/742	-	-	ν CC (5-mem.),
-/786	-	769	-	ν CC (5-mem.), γ CH + γ COH
812/816	829	-	829/830	CC, NH_2, γ NH + γ CCN
851/854	860/860	851/852	-	CH wag, γ NH + γ CCN, ZnO comb. mod.
878	887	-	-	CH_2 rock
928/928	947/-	-	932/932	ν CC (5-mem.), γ NH + γ CCN
1007/-	-	1000/ 998,	997/997	γ =CH + γ CCC, ZnO (A_1)
1022/1025	1018/-	1026/1017	1024/1017	

	1033/1032	1023/1024		γ CH, ρ (OH) + (CH) + (NH2), ZnO comb. mod.
1041/1045	1050/1050	1053/1054		γ CH, ν CC, ZnO (A_1)
1083/1089	1096/1096	1077/1091,1118	1079/1089,1116	γ CH, ν CO, δ CC, ZnO comb. mod.
1159/1163	1169/1171	-	1157/-	ν C=C + δ CH, ZnO comb. mod.
-	-	-	1181/-	δ CH + δ COH
1212/1215	1222/1222	-	-	δ CH + δ CCH
-	-	1294/1256	1294/1294	
1316/1297	1326/1304	-/1316	-	
-	-	1345/1345	1347/-	
1387/1391	1396/1391	-	-	δ NH + δ CH
-	-	1421/1413	1420/1410	σ HCH
1442/1446	1439/1435	-	-	ν C=C + δ =CH
-	1490/1490	1471/1464	1473/1465	
-	-	1555/1565	1554/1566	
1588/-	1597/-	-	-	ν C=C + δ CH
1602/1604	-	1601/1600	1601/1601	σ NH2
-	1615/1612	-	-	ν C=C + ν C-O

***ν: stretching, δ: in-plane bending, γ: out-of-plane bending, ω: wagging, ρ: rocking, σ: scissoring, comb. mod.: combination modes. All OH vibration modes are assignment based on the literature, however in our case it is oxygen atom from ligand bonded to ZnO surface.**

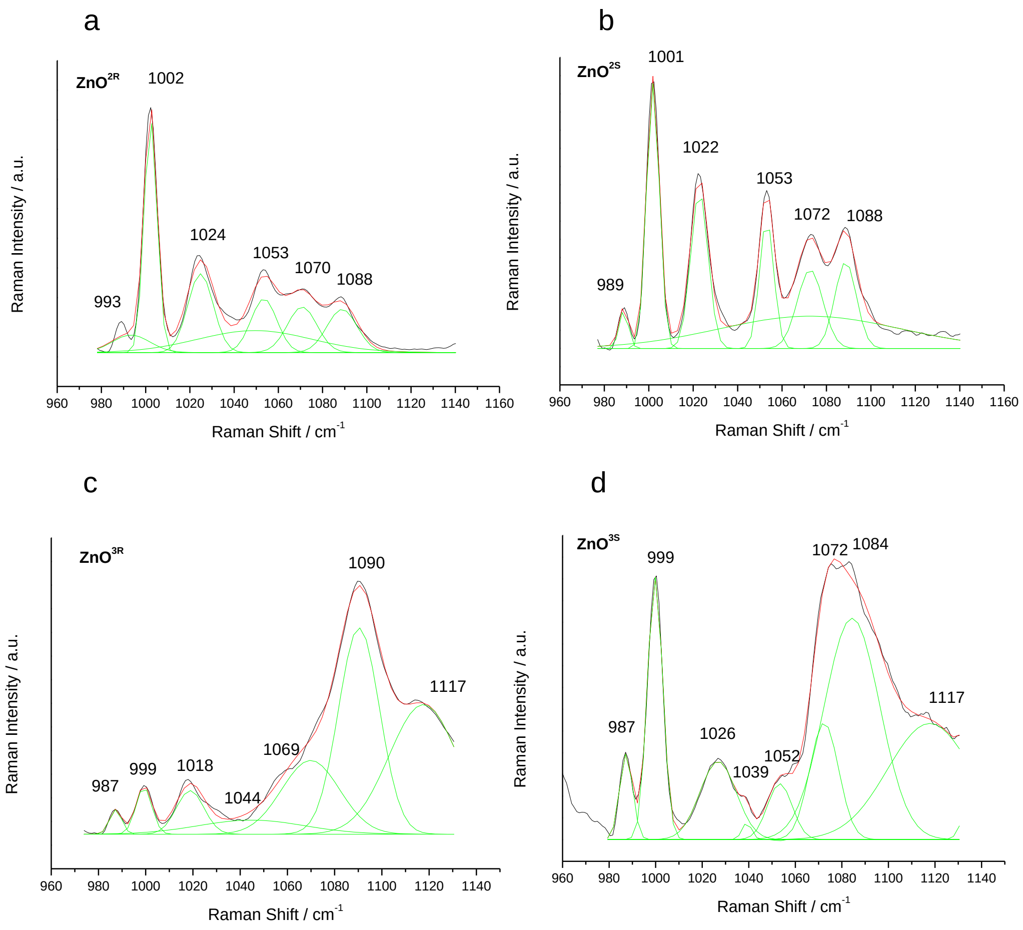

Figure S23. The Raman spectra in the 960-1160 cm^{-1} region collected for **ZnO^{2R}** (a), **ZnO^{2S}** (b), **ZnO^{3R}**(c), **ZnO^{3S}** (d). For the assignment see Table S1.

The component principal analysis

The component principal analysis(PCA) was performed over the pre-processed Raman spectra. First, Raman spectra were smoothed with Savitsky-Golay filter, the background was removed using baseline correction (10 itenary and 64 points), and then the spectra were normalized using a so-called Min-Max normalization using a built-in OPUS software package (Bruker Optic GmbH 2012 version). After that, the data were transferred to the Unscrambler software (CAMO software AS, version 10.3, Norway), where the PCA calculation was performed based onto the NIPALS algorithm, validation (random with 20 segments), significance 0.05 and the 90 number of samples (Raman spectra).The presented below data are based on the PCA analysis for the regions of200-2000 cm^{-1} wavenumbers.

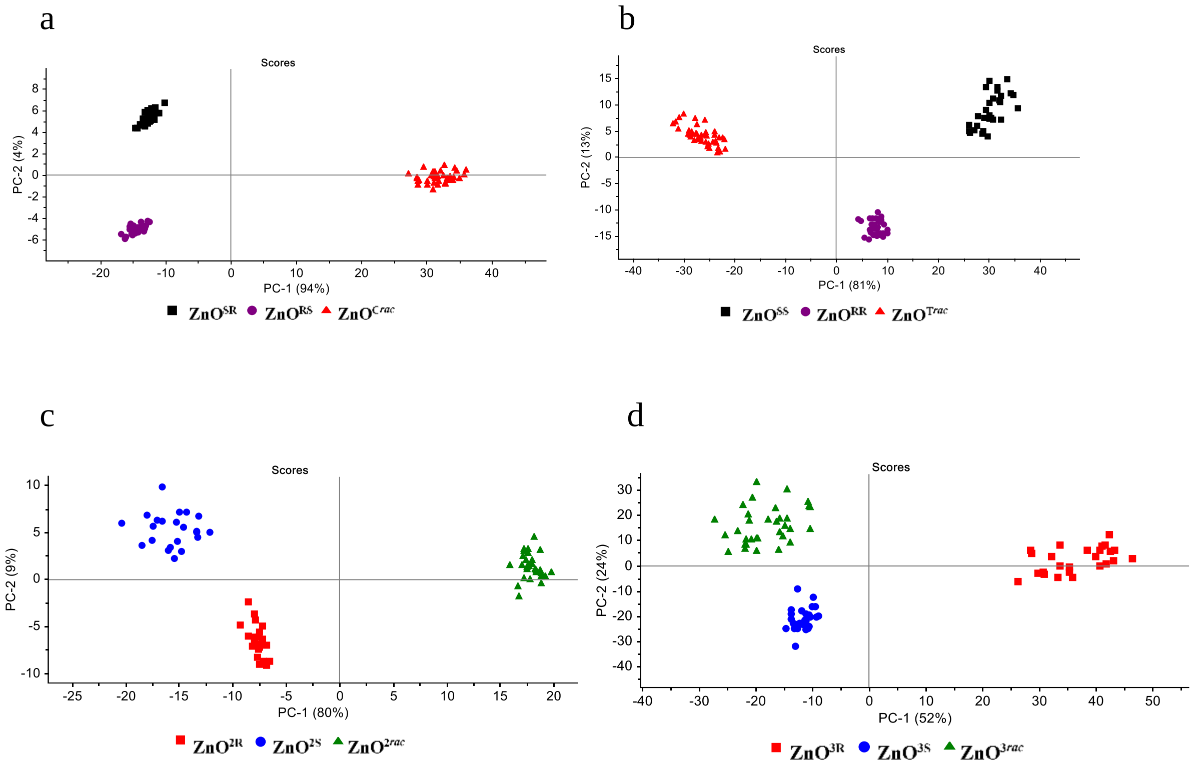

Figure S24. PCA scores of **ZnO^{1SR}**, **ZnO^{1SR}**, **ZnO^{1Crac}** (a); **ZnO^{1RR}**, **ZnO^{1SS}**, **ZnO^{1Trac}** (b); **ZnO^{2R}**, **ZnO^{2S}**, **ZnO^{2rac}** (c); **ZnO^{3R}**, **ZnO^{3S}**, **ZnO^{3rac}** (d). All calculation made in the 200 - 2000 cm^{-1} region.

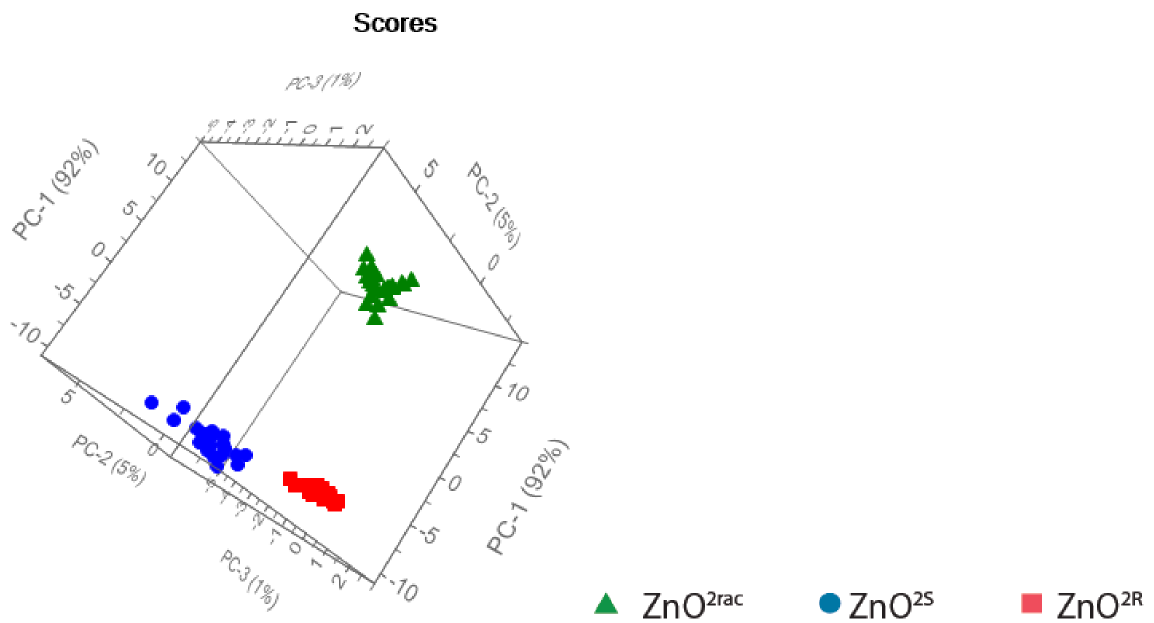

Figure S25.PCA scores of **ZnO^{2R}**, **ZnO^{2S}**, and **ZnO^{2rac}**. For 960 – 1120 cm^{-1} range.

Table S3. Calculated the weighted variables (absolute values) in the loading of PC1 and PC1 + PC2 scores for the 960-1120 cm^{-1}region.The most weighted variables are bolded.

Analyzed groups of ZnO NCs	Variable	weighting	PC1 + PC2 [%]
ZnO^{1RS}, ZnO^{1SR}, ZnO^{1Crac}	1018	0.08	**99**
	1024	0.16	
	1033	0.03	
	**1043**	0.30	
	1057	0.02	
ZnO^{1RR}, ZnO^{1SS}, ZnO^{1Trac}	1007	0.01	**97**
	1012	0.10	
	**1022**	0.32	
	1032	0.08	
	1038	0.06	
	1050	0.02	
	1083	0.06	
Zn^{2R}, ZnO^{2S}, ZnO^{2rac}	1006	0.07	**97**
	1012	0.05	
	**1022**	0.43	
	1040	0.02	
	1048	0.07	
	1074	0.05	
ZnO^{3R}, ZnO^{3S}, ZnO^{3rac}	**1000**	0.29	**87**
	1014	0.003	
	1032	0.10	
	1054	0.003	
	1089	0.13	
ZnO^{1RR}, ZnO^{1SS}, ZnO^{1RS}, ZnO^{1SR}	1006	0.08	**96**
	1012	0.09	
	**1022**	0.32	
	1033	0.05	
	1042	0.12	
	1082	0.04	

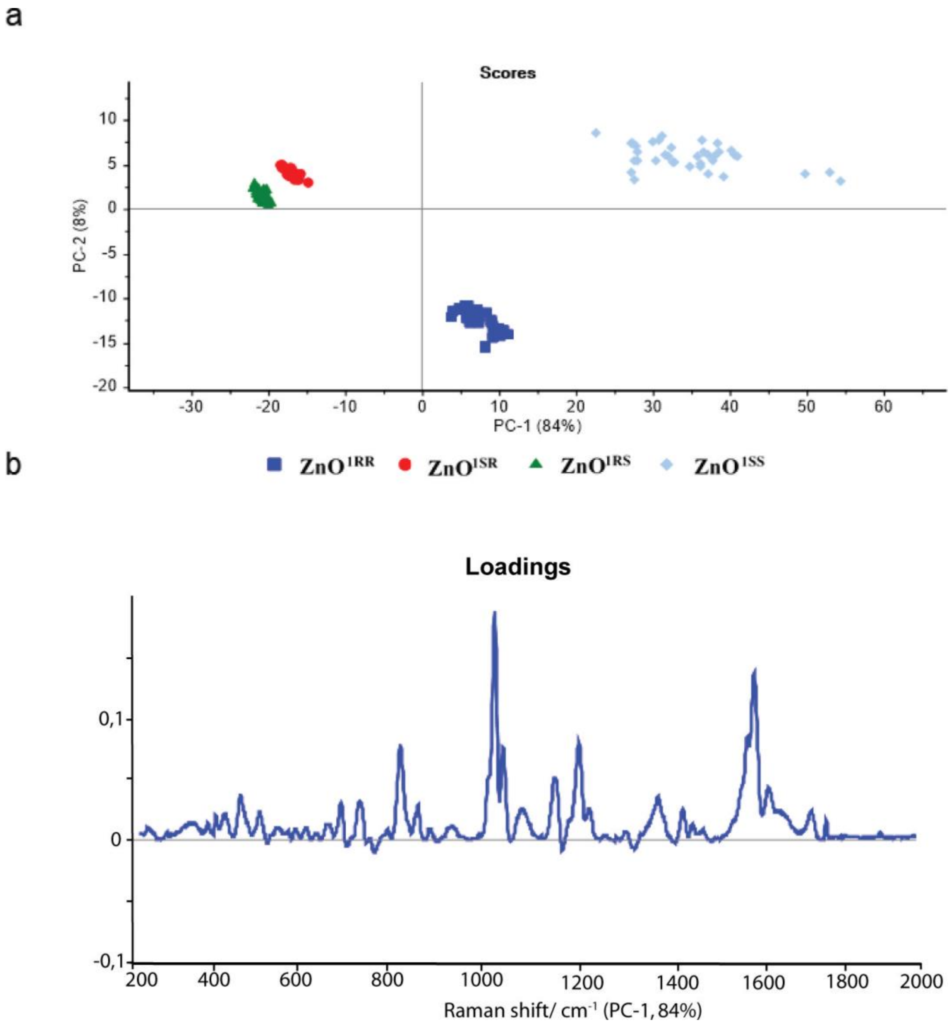

Figure S26. Scheme presents the PCA classification of **ZnO^{1SS}**, **ZnO^{1RR}**, **ZnO^{1SR}** and **ZnO^{1RS}**, (a) and the loadings (b) performed for 200 - 2000 cm^{-1} region.

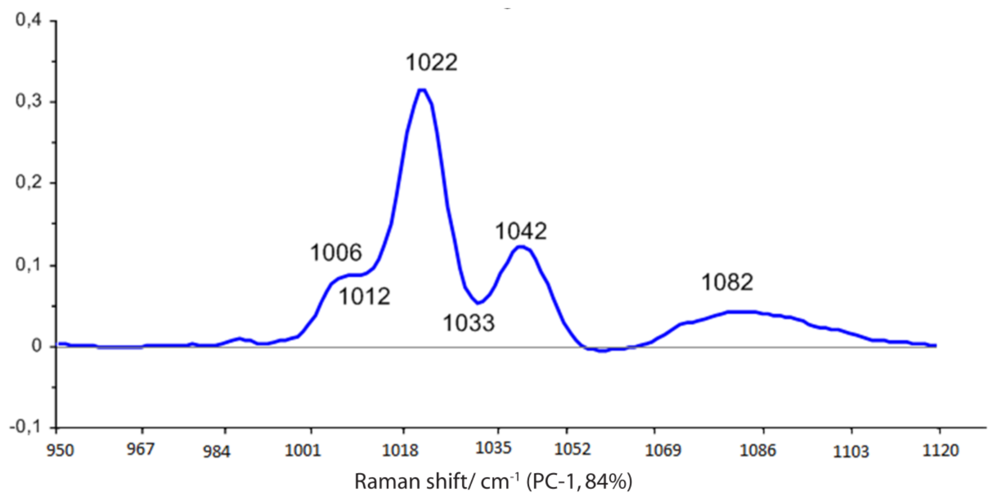

Figure S27. PC1 loadings for **ZnO^{1SS}**, **ZnO^{1RR}**, **ZnO^{1SR}**, **ZnO^{1RS}** . Presented calculations are taken in 960 – 1120 cm^{-1} region.

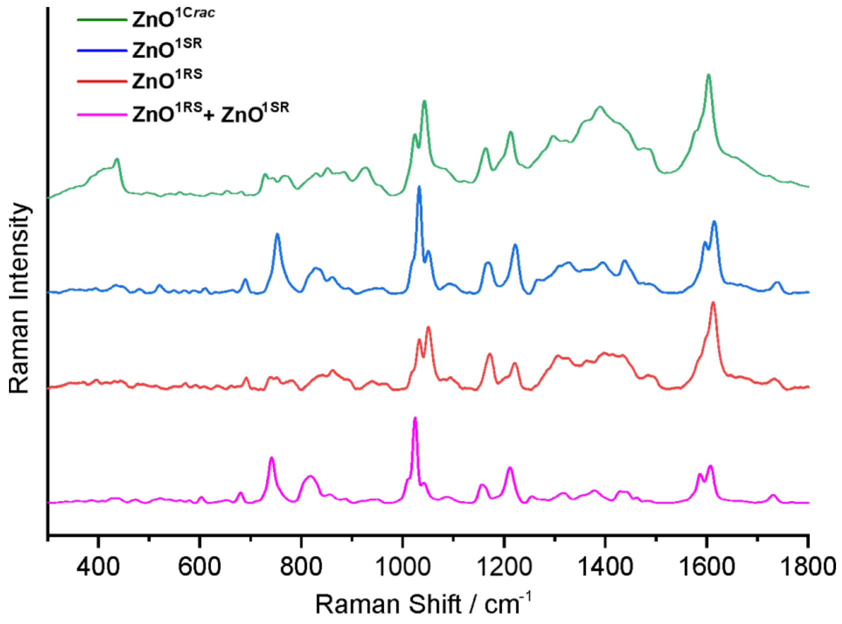

Figure S28. The Raman spectra of **ZnO^{1Crac}**, **ZnO^{1SR}**, **ZnO^{1RS}** and the mixture of **ZnO^{1RS}** and **ZnO^{1SR}**.

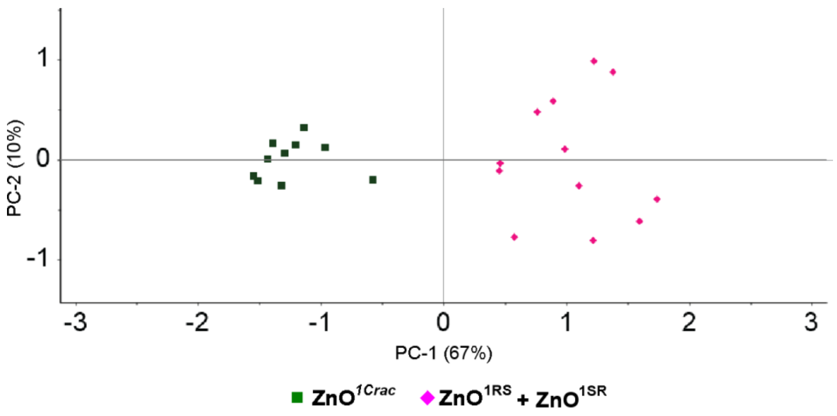

Figure S29. PCA scores of **ZnO^{1rac}** and a mixture of **ZnO^{1RS}** and **ZnO^{1SR}**. For 200 – 2000 cm^{-1} region.

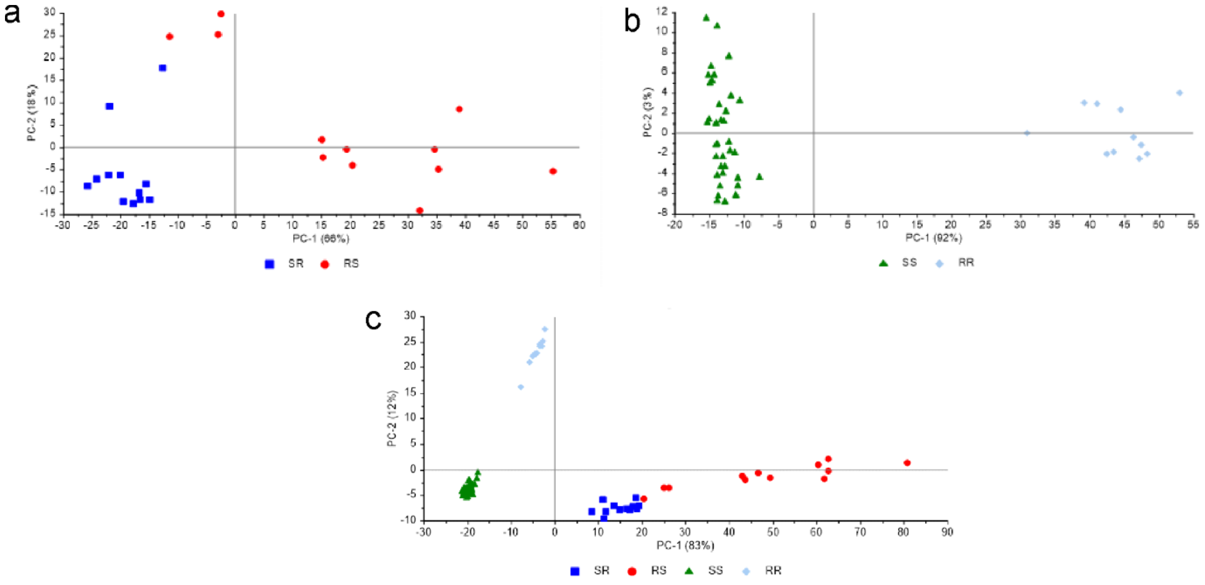

Figure S30. PCA scores of (1R, 2S)-, (1S, 2R)-*cis*-1-amino-2-indanol (**RS, SR**) (a), (1R, 2R)-, (1S, 2S)-*trans*-1-amino-2-indanol (**SS, RR**) (b), and (1R, 2S)-, (1S, 2R)-, (1R, 2R)-, (1S, 2S)-1-amino-2-indanol (**RS, SR, SS, RR**) (c).

REFERENCES

(1) Chwojnowska, E.; Wolska-Pietkiewicz, M.; Grzonka J.; Lewiński, J. Anorganometallicroute to chiropticallyactiveZnOnanocrystals. *Nanoscale* **2017**, *9*, 14782 – 14786.

(2) K.; Subashini, S.; Periandy. Spectroscopic (FT-IR, FT-Raman, UV, NMR, NBO) investigation and molecular docking study of (R)-2-Amino-1-PhenylEthanol. *Journal of Molecular Structure***2016**, *1117*, 240-256.

(3) Hiroshi Iga, TasukuIsozaki, Tadashi Suzuki, and TeijiroIchimura. Conformations of 2-Aminoindan in a Supersonic Jet: The Role of Intramolecular N-H,,,π Hydrogen Bonding. *J. Phys. Chem. A***2007**, *111*, 5981-5987.
(4) Collaboration: Authors and editors of the volumes III/17B-22A-41B. Zinc oxide (ZnO) phonon wavenumbers: combination modes. In: Madelung O., Rössler U., Schulz M. (eds) II-VI and I-VII Compounds; Semimagnetic Compounds. Landolt-Börnstein - Group III Condensed Matter (Numerical Data and Functional Relationships in Science and Technology), vol 41B. Springer, Berlin, Heidelberg.
